# Accurate and Fast Simulation of Channel Noise in Conductance-Based Model Neurons by Diffusion Approximation

**DOI:** 10.1371/journal.pcbi.1001102

**Published:** 2011-03-10

**Authors:** Daniele Linaro, Marco Storace, Michele Giugliano

**Affiliations:** 1 Biophysical and Electronic Engineering Department, University of Genoa, Genoa, Italy; 2 Department of Biomedical Sciences, University of Antwerp, Wilrijk, Belgium; 3 Laboratory of Neural Microcircuitry, Brain Mind Institute, EPFL, Lausanne, Switzerland; 4 Department of Computer Science, University of Sheffield, Sheffield, United Kingdom; Université Paris Descartes, Centre National de la Recherche Scientifique, France

## Abstract

Stochastic channel gating is the major source of intrinsic neuronal noise whose functional consequences at the microcircuit- and network-levels have been only partly explored. A systematic study of this *channel noise* in large ensembles of biophysically detailed model neurons calls for the availability of fast numerical methods. In fact, exact techniques employ the microscopic simulation of the random opening and closing of individual ion channels, usually based on Markov models, whose computational loads are prohibitive for next generation massive computer models of the brain. In this work, we operatively define a procedure for translating any Markov model describing voltage- or ligand-gated membrane ion-conductances into an effective stochastic version, whose computer simulation is efficient, without compromising accuracy. Our approximation is based on an improved Langevin-like approach, which employs stochastic differential equations and no Montecarlo methods. As opposed to an earlier proposal recently debated in the literature, our approximation reproduces accurately the statistical properties of the exact microscopic simulations, under a variety of conditions, from spontaneous to evoked response features. In addition, our method is not restricted to the Hodgkin-Huxley sodium and potassium currents and is general for a variety of voltage- and ligand-gated ion currents. As a by-product, the analysis of the properties emerging in exact Markov schemes by standard probability calculus enables us for the first time to analytically identify the sources of inaccuracy of the previous proposal, while providing solid ground for its modification and improvement we present here.

## Author Summary

A possible approach to understanding the neuronal bases of the computational properties of the nervous system consists of modelling its basic building blocks, neurons and synapses, and then simulating their collective activity emerging in large networks. In developing such models, a satisfactory description level must be chosen as a compromise between simplicity and faithfulness in reproducing experimental data. Deterministic neuron models – i.e., models that upon repeated simulation with fixed parameter values provide the same results – are usually made up of ordinary differential equations and allow for relatively fast simulation times. By contrast, they do not describe accurately the underlying stochastic response properties arising from the microscopical correlate of neuronal excitability. Stochastic models are usually based on mathematical descriptions of individual ion channels, or on an effective macroscopic account of their random opening and closing. In this contribution we describe a general method to transform any deterministic neuron model into its *effective* stochastic version that accurately replicates the statistical properties of ion channels random kinetics.

## Introduction

Ion channels are the fundamental elements underlying neuronal excitability and information transfer, inter- and intracellularly. These protein pores, found also in other excitable cell types, undergo fast conformational modifications (hereafter referred to as *channel gating*) induced by a change in the electric field or by the binding of ligand molecules. By doing so, channels selectively affect the ionic conductances of the membrane and enable ions to flow according to their electrochemical potentials [1]. The impact of the first quantitative deterministic description of conductance gating [2] was extremely significant, as testified by its wide use up to today [3]. Since the 1970s however, the stochastic nature of the single ion channels gating has been fully recognised. The resulting random fluctuations in the membrane conductances (which are known as *channel noise*) have been the subject of intense theoretical and experimental research [4]–[12]. Nevertheless, only recently *channel noise* was emphasised to have a significant impact on neuronal signals generation, propagation and integration, and it was suggested for consideration in realistic models of single neurons [13]–[19]. In some parts of the peripheral nervous system, *channel noise* has been demonstrated to be prominent for information transfer and perception (e.g., see [20] and references therein). However, in the central nervous system whether or not *channel noise* plays a role at the level of large networks of interacting neurons, how heterogeneous ion channel types contribute to spontaneous network firing, and whether *channel noise* combines or interferes with other sources of noise (synaptic, for instance) remain open questions.

Towards addressing these questions, the increasing availability of cheap parallel computing resources and improved algorithms [21], [22] allow one to approach *in silico* the study of networks of thousands of morphologically detailed multi-compartmental model neurons [23]. In addition, a diversity of voltage- and ligand-gated ion channel types can be included in these large models with biophysical realism [24]. Unfortunately, *channel noise* is rarely considered for large network simulations or detailed multi-compartmental models [25], due to its heavy computational load. Implementing single-channel stochastic models explicitly, for each of the thousands of channels per ion conductance type and per neuron, requires Montecarlo simulation techniques [5], [14], [15], [26] that are computationally intensive even for single compartmental neurons, regardless of excellent speed-up techniques [14]. Throughout this paper, we refer to such explicit and *exact* simulation methods by the term *microscopic*, regardless of the details of their actual numerical implementation [27].

For the specific case of the Hodgkin-Huxley (HH) equations, Fox and collaborators proposed an alternative approximate method to mimic *channel noise*, avoiding a microscopic description of the individual channels [28], [29]. This method relies on the use of stochastic differential equations to *macroscopically* account for the fluctuations in the overall conductance of sodium and potassium channels, with formal analogies to the Langevin equation [14], [30]. Although this approach is very attractive and was employed widely in the literature (see references in [31]), its accuracy was recently challenged and debated by several authors [27], [31]–[33]. These authors compared numerical simulations of the exact microscopic descriptions of the HH model with those obtained by Fox's, finding some inconsistencies. It was however only with the work by Bruce (2009), that a straightforward test and framework were proposed to quantify the accuracy of Fox's algorithm. Simulating a *voltage-clamp* experiment, while recording ion currents, clearly shows that Fox's approximation does not capture correctly the microscopic statistical properties, regardless of how large the number of single ion channels to be approximated is. An *ad hoc* partial correction of Fox's algorithm - based on the simultaneous Montecarlo simulations of single channels - was also proposed for some activity regimes [31], but it cannot be generalised to arbitrary simulation conditions.

In this paper we introduce and operatively define a general method, based on the diffusion approximation [30], to transform any deterministic model neuron into its *effective* stochastic version, for an arbitrary set of ion conductances. As in previous studies, we focus on discrete Markov processes [8], [34], routinely employed in the experimental identification of voltage-gated channels and synaptic receptors. Our purpose is to reintroduce channel noise in deterministic conductance-based models with limited computational overhead. We also aim at accurately replicating the statistical properties of ion conductances, as predicted by the exact microscopic description, while avoiding the use of any *ad hoc* correction or heuristics in the choice of the parameters [35]. Our approach is related to previous Langevin-based formulations, although with a significant difference in the way channel fluctuations are reintroduced in model equations. It can be considered as an accurate and systematic generalisation of Fox's algorithm, to the case of voltage-, ion-, and ligand-gated channels with arbitrary complexity. We numerically compare our approach to that by Fox and we provide, as a Supporting Information, some analytical results showing where it fails. We validate our approach for single-compartmental neuronal simulations, incorporating HH fast inactivating sodium channels and delayed rectifier potassium channels, analogously to previous works. By comparing our effective method to the exact simulations of the stochastic channel kinetic schemes, we obtain satisfying agreement.

## Materials and Methods

In this section, we briefly review the deterministic HH model and then introduce our algorithm. We present our method for ion channels whose microscopic correlate is represented by a population of identical 2-state channels. Only in this specific case, our method coincides with Fox's approach. We then generalise the method to channels characterised by 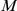-state kinetics and show that, for the special case of multiple independent subunits, each composed by 2-state gating mechanisms as in HH-like currents, the mathematical expressions underlying our algorithm greatly simplify.

### Neuron model

**Figure 1:**
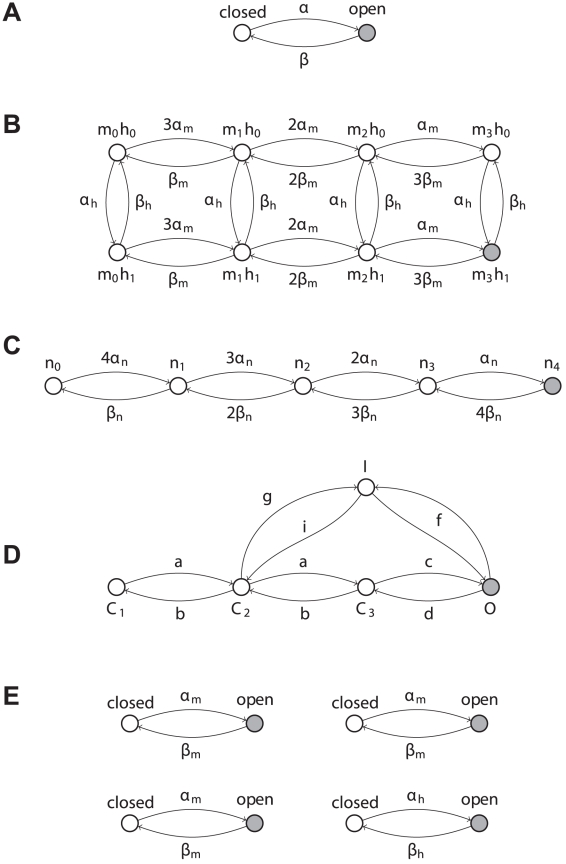
Markov kinetic schemes. In the simplest 2-state kinetics (**A**), a single channel can be in one of two configurations with only one of them associated to a non-zero conductance (filled grey circle). The kinetic parameters 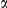 and 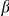 are rates, as they represent the transition probabilities between states, expressed per time unit. In a more general case, single-channel kinetics is described by an 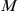-state scheme. Voltage-gated fast-inactivating sodium (**B**) and delayed-rectifier potassium channels (**C**) are two examples, where only one state corresponds to a non-zero channel conductance (filled grey circle). An alternative model for sodium channels (**D**) (Vandenberg and Bezanilla, 1991) is also shown for comparison. We point out that our method can be applied to any kind of kinetic schemes, where the transition rates are known. For (**B–C**), each state is identified by an arbitrary name convention (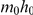, 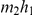, 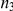, etc.), referring to the underlying mapping of these 8- and 5-state channels into multiple 2-state gated subunits (panel **E**). Indeed, some 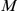-state kinetic schemes may be mapped into, or experimentally identified as, a set of independent 2-state gates: the open state of the full scheme corresponds to all the elementary gates in the open states, simultaneously. For instance, the kinetic scheme (**B**) could be mapped into a set of four independent 2-state gates (**E**) (i.e., the familiar *activation* gates and the *inactivation* gate of sodium fast-inactivating currents), three of whom are identical.

We consider a single-compartmental conductance-based neuron model [36]. For this class of models, the membrane potential 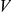 obeys the following current balance equation [1]

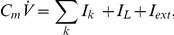
where 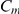 is the specific membrane capacitance and 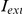 is an externally applied current density (expressed in 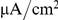). These models comprise a leak current 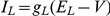 and a number of intrinsic (as well as synaptic) currents that can be similarly expressed by an *ohmic* relationship 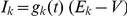, which links the current to the membrane potential. Each ionic conductance 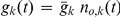 is completely determined by the fraction of corresponding channels 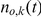 in the *open* state (see Fig. 1A–D).

For reference to previously published papers [9], [10], [12], [15], [18], [29], we consider here the HH voltage-gated currents 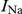 and 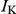 with standard parameters [2]. Therefore, we consider 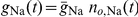 and 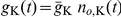. In the deterministic model, 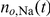 and 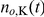 are expressed phenomenologically as a product of activation and inactivation deterministic variables [37]–[40]:
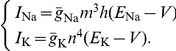
Each of these variables obeys a first-order ordinary differential equation of the form 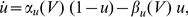
(1)where 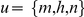 and 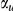, 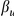 are kinetic parameters. All the model parameters are summarised in Table 1.

**Table 1:** Parameters employed for the deterministic simulations.

Symbol	Description	Value
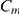	Membrane capacitance	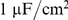
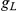	Leak conductance	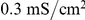
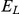	Leak reversal potential	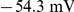
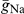	Max sodium conductance	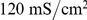
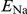	Sodium reversal potential	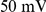
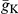	Max potassium conductance	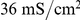
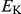	Potassium reversal potential	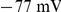
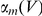	Kinetic parameter of 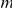 gates	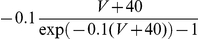
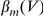	Kinetic parameter of 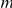 gates	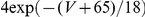
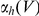	Kinetic parameter of 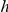 gates	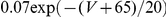
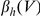	Kinetic parameter of 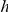 gates	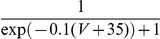
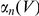	Kinetic parameter of 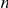 gates	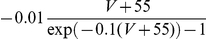
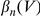	Kinetic parameter of 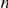 gates	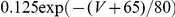

### Exact simulation of the microscopic models

Montecarlo methods represent the most commonly adopted way to simulate the random temporal evolution of ion conductances in a membrane patch, populated by a set of identical independent channels. Due to spatial proximity, channels are assumed to be coupled together by a common gating variable, such as the membrane potential or the local neurotransmitter concentration. Then, the full knowledge of the Markov kinetic scheme (see Fig. 1A–D) describing the distinct conformational states of each ion channel, as well as the transition probabilities across states, are needed [41], [42]. The kinetic scheme is employed to simulate the random transitions of the state of each individual ion channel, by repeated pseudo-random number generation (see [5], [14], [15], [26] and references therein). Although refined fast-computation techniques have been proposed [14], we employ here a basic numerical implementation. Briefly, instead of tracking the state of each channel, the number of channels in a given state is tracked and updated at each time step (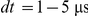), with conditional probabilities that depend on the transition rates of the Markov scheme, as exemplified in Fig. 1A. We recall that simulating the occurrence of a random event with probability 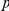 can be achieved by generating a pseudo-random number 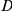, uniformly distributed between 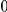 and 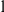, and testing whether or not 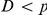 [43]. In the simulations reported here, we set the single-channel conductance for both sodium and potassium channels to 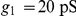, unless specified otherwise, and we consider a fixed channel density of 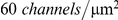 and 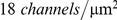 for sodium and potassium currents, respectively. In all simulations, a cylindrical single compartment was used with length and diameter equal to 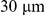, unless otherwise noted. Albeit conceptually simple, these algorithms require a great amount of computational power, which increases with the number of channels that are to be simulated and with the probability of their activation. Simulation code and analysis scripts, developed in C++ and in NEURON [44], are available from ModelDB [45] at http://senselab.med.yale.edu/modeldb via accession number 127992.

### Population of two-state channels

We examine the case of a ion current whose microscopic correlate is represented by a population of 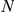 ion channels. The single-channel kinetics is a 2-state scheme: *open* and *closed*, as shown in Fig. 1A. This is the simplest kinetic scheme and is often employed, for instance, for the minimal description of ionotropic AMPA-receptors [46]. The symbols 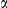 and 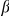 in Fig. 1A represent the transition probabilities between states, expressed per time unit (i.e., as rates). They are functions of the channel gating variable(s) – such as membrane voltage, intracellular calcium concentration, extracellular magnesium concentration, extracellular glutamate concentration, etc. [8] – and are experimentally identified by routine electrophysiological techniques [7] and optimisation methods [34].

For the definition of our effective simulation technique for *channel noise*, we consider five realistic assumptions: (i) the channels are identical and statistically independent; (ii) for simplicity, only one conformational state is associated to a non-zero ion conductance 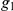; (iii) 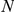 is large and is known; (iv) the single-channel kinetics is described by a Markov process, where transition probabilities depend only on the current state and on the gating variable(s), and not on the previous occupancy history; and (v) the gating variables (e.g., 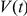) change slowly, compared to the channel kinetics, with time constant 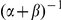 [1].

Because of (i)–(ii), the maximal ion conductance associated to the channels can be expressed as 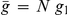. Then, the time-varying conductance 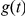 depends only on 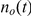, the fraction of channels in the *open* state:
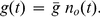
(2)Since individual channels undergo random transitions between states [7], 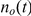 is a non-stationary random variable, whose instantaneous value is distributed according to a binomial probability function: the number of open channels, 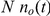 (with 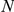 constant), is a binomial random variable. As a consequence, the statistical properties of 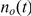 are fully specified by 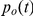, the probability of occupancy of the *open* state [6]. By assumption (iii), the binomial distribution of 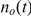 can be approximated by a Gauss distribution, invoking the de Moivre-Laplace (or central limit) theorem, valid when 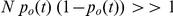 [47]. By (iv), 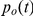 can be numerically computed as the solution of the following linear differential equation [6], formally equivalent to the deterministic kinetic Eq. 1 [48]:
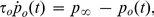
(3)with 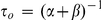 and 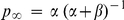. Finally, under assumption (v), Eq. 3 can be solved analytically and 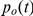 is expressed as an exponential function. Under these approximations, 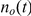 is Gauss-distributed and completely described by its mean 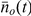 and by its (auto)covariance function 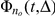, which at the steady-state has an exponentially decaying profile: 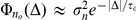 [6], [49]. In the theory of stochastic processes, 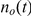 is called a *diffusion* process, with 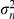 and 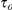 its steady-state variance and autocorrelation time constant, respectively [30].

By these considerations, it follows that 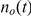 can be approximated and computer-simulated by an efficient method, alternative to the exact Montecarlo simulation of the discrete kinetic scheme [14]. This method consists in generating a realisation of an Ornstein-Uhlenbeck's process [30], with time-varying mean 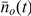, steady-state variance 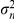, and autocorrelation time constant 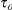:
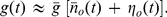
(4)

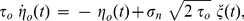
(5)where 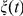 is a 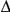-correlated Gauss-process with zero mean [47] (see also Eq. 20).

Since 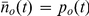 [6], [49], the deterministic component of 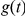 evolves as Eq. 3, which is the familiar equation one expects by the mass-action law (i.e., Eq. 1), while interpreting as deterministic the scheme of Fig. 1A [2], [38]. For clarity, we rewrite such an equation as 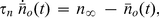
(6)with 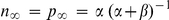, and 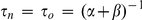.

As opposed to the deterministic HH formalism however, the stochastic nature of 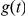 is now explicitly captured by 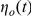, algorithmically generated as a pseudo-random process by iterating the discrete-time version of Eq. 5 [50], reported for the sake of completeness in Eqs. 23–24. Thus, by setting 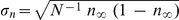, Eqs. 4, 5, and 6 reproduce both the time-varying mean and the steady-state covariance of 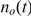. More precisely, 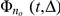 and the covariance of the term 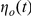 relax to the same analytical expression 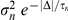, after a transient of the order of 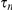.

Finally, the clipping of negative conductance values for 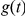 may be necessary but, if lacking, it will not affect by accumulation the numerical integration of 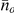 in the present form of Eq. 4.

We remark that we do not (heuristically) add a noise term in the right-hand-side of Eq. 6, as in previous Langevin-based algorithms. Instead, a precise approximation procedure is employed to statistically mimic the effect of channel noise fluctuations in 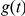. Although for 2-state channels Eqs. 4–6 are indeed equivalent to Fox's formulation (see the Text S1), our approach differs considerably from that by Fox as soon as multiple-state channels are considered, e.g., the sodium fast-inactivating and the potassium delayed-rectifier channels.

### Population of *M*-state channels

We now generalise the diffusion approximation (Eqs. 4–6) to the more general case of a large population of identical independent channels, whose single-channel dynamics is described by an *M*-state Markov scheme. Under the same assumptions (i)–(v), the probability 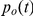 of occupancy of the open state fully describes the fraction of open channels (see Eq. 2). However, now 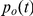 is a particular (say, the *k-*th) element of the 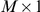 probability vector 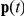 of state occupancy, and each element of 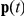 corresponds to a distinct state of the kinetic scheme. By assumption (iv), 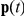 satisfies a system of *M* linear ordinary differential equations, which can be written in compact form as
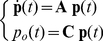
(7)The 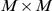 transition matrix 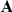 is filled with the appropriate combinations of the individual transition rates between all the possible states [51]. 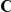 is a 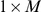 vector with only one (the *k-*th) non-zero element, set to 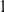. Under assumption (v), 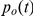 can be computed analytically as a linear combination of a steady-state value 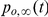 and of *M-1* exponentials with time constants 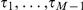, each being the inverse of the absolute value of a non-zero eigenvalue of 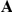 [51]. As for the 2-state kinetics, the statistical properties of the fraction of open channels 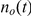 are fully specified by 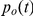 and by the binomial distribution [6]. By assumption (iii), the distribution of 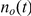 can be approximated by a Gauss-distribution [47], and 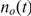 can be numerically simulated by an equivalent diffusion process. However, differently from the previous case, the steady-state covariance 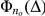 contains a weighted sum of *M-1* exponentials [6], [49] and not a single term:
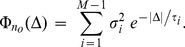
(8)Therefore, Eq. 4 no longer approximates 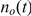, and it must be extended to a linear combination of *M-1* Ornstein-Uhlenbeck's independent processes 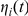, with appropriate coefficients and time constants: 
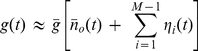
(9)

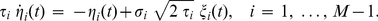
(10)As for the 2-state model, 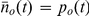. Then, one always recovers the deterministic description of the 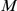-state channels, formally coincident with Eq. 7. The derivation of the analytical expressions for 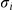 and 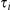 is necessary, as they depend on the values of the gating variable(s) (e.g., 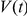), and requires the full expression of 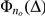 [6], [49],
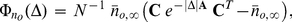
(11)which can be obtained by Laplace-transforms or linear algebraic methods [52].

We remark that, for our purposes, the derivation of Eq. 11 is important mainly to introduce Eqs. 8–10. Indeed, Eq. 11 considerably simplifies in the case of ion channels whose 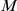-state kinetics can be mapped into, or have been experimentally identified as, the composition of several 2-state subunits. For instance, the scheme of Fig. 1B can be mapped into the equivalent kinetic scheme shown in Fig. 1E. This is very common in the computational neuroscience literature for voltage- and ligand-gated ion channels, whose single-channel open state corresponds to the simultaneous active state of a multiple number of independent subunit types. To illustrate how Eq. 11 simplifies, we discuss a specific example where three different subunit types are present [37], [38], although our considerations hold for any number of different subunit types. We name these three subunit types as *m*, *h*, and *s*, and for each of them we compute the steady-state probabilities of the active state and the gating time constants, following from the solution of Eq. 3:
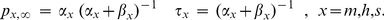
(12)We further assume that the overall single-channel conductance results from the composition of a given number of elements of each subunit type: say, *q*, *r*, and *w* subunits of the type *m*, *h*, and *s*, respectively. For instance, in the kinetic scheme of Fig. 1E, we have 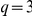, 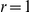, and 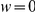. Since each subunit is described by 2-state kinetics, the total number *M* of states is 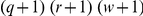. By this definition, the process 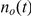 is binomial and described by the joint probability that all subunits are simultaneously in their open state. Because of the statistical independence of each subunit, the joint probability is the product of elementary probabilities [6].

Under the same assumptions of previous section, 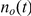 can be approximated by a diffusion stochastic process, combining deterministic and stochastic terms, as in Eq. 4. Being 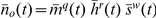, we can rewrite Eq. 9 as follows: 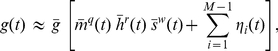
(13)

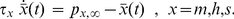
(14)Since in this case the covariance of a product is the product of covariances, Eq. 11 reduces to [6], [49]

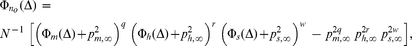
(15)with 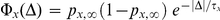, and 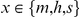. Expanding the powers and products of Eq. 15 and obtaining the expressions for the 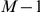 distinct coefficients 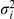 and time constants 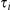, needed for Eqs. 9 and 10, is easier than manipulating the matrix exponential of Eq. 11.

In the specific case of HH fast-inactivating sodium (i.e., 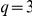, 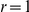, and 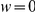) and delayed rectifier potassium channels (i.e., 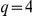, and 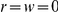) (Fig. 1B–C), 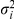 and 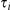 take the expressions reported in Table 2.

**Table 2:** Values of the coefficients 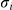 and of the time constants 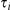 for fast-inactivating sodium and delayed-rectifier potassium channels to be used in Eqs. 9–10.

Coefficient	Sodium	Potassium	Time constant	Sodium	Potassium
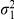	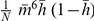	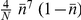	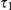	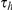	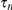
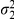	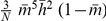	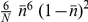	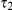	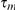	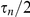
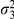	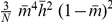	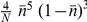	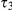	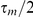	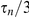
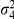	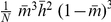	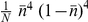	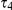	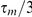	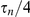
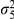	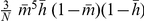	–	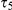	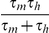	–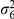	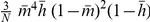	–	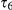	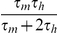	–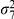	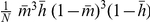	–	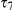	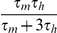	–

The steady-state symbol (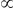) was omitted for the sake of notation, from all occurrences of 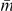 and 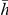.

### Approximate reduction to a single noise term

In order to further gain in computational efficiency, while numerically implementing our diffusion approximation of *channel noise* (Eqs. 9–10), it is possible to reduce to one the number of required independent Ornstein-Uhlenbeck's stochastic processes. This additional approximation consists in interpolating the covariance of 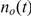 by a single decaying exponential, by replacing Eq. 9 with Eq. 4. Indeed, since Eq. 8 is the weighted sum of 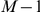 exponentials, one should not privilege any of those terms *a priori* and appropriately choose 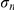 (in Eq. 4) and 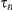 (in Eq. 5) as best-fit parameters for each value of the gating variable(s), so that
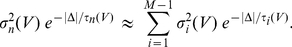
(16)


Alternatively, by expanding both sides of Eq. 16 by the Taylor series, extended to the first-order (or higher), the dominant term around 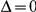 can be approximated by setting
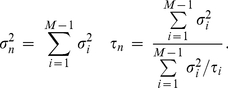
(17)


In investigating the impact of *channel noise* on the computational properties of single-neurons and networks, such a systematic and controlled reduction procedure should replace heuristic methods and may be extremely useful to dissect whether or not each of the 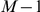 terms is needed in accounting for a particular observation.

### The complete effective model

Following Eqs. 9–10 and Table 2, we now formulate the effective stochastic model, corresponding to the deterministic HH model introduced earlier:
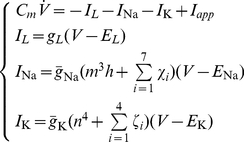
(18)


The deterministic gating variables 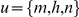 still obey Eq. 1, while each of the 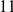 new stochastic variables (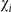 and 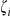) is described by Eqs. 9 and 10:
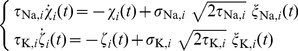
(19)where 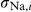, 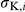, 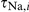, and 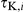 are the coefficients given in Table 2, while 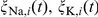 are independent, identical, 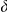-correlated, Gauss-distributed processes with zero means and unitary variances (see Eqs. 23–24).

We emphasise that the procedure leading to Eq. 18 is general and can be easily applied to more complex (single- and multi-compartmental) neuron models, which incorporate arbitrary ionic currents.

### The Ornstein-Uhlenbeck's stochastic process

Since the Ornstein-Uhlenbeck's stochastic process has been referred to repeatedly in the previous sections, we concisely review its definition and its practical numerical simulation. A realisation of this process, say 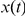, can be operatively defined as the exponential filtering of a Gauss-distributed white noise. Abusing the notation of ordinary differential equations, 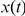 is the solution of
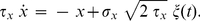
(20)The term 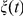 represents a stationary Gauss-distributed stochastic process, which is a white-noise, fully specified by its mean 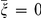 and covariance 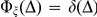.

By linearity, 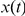 is also Gauss-distributed [47] and characterised by non-stationary mean 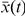 and covariance 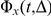:
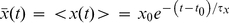
(21)

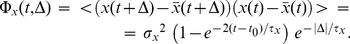
(22)These quantities converge to stationary values after a time of the order of 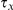, so that at the steady-state 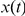 has mean and variance equal to zero and 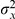, respectively, and an exponentially-decaying autocorrelation function, with time constant 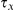.

For the purpose of obtaining independent realisations of 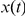 in computer simulations, a discrete-time equivalent of Eq. 20 must be employed to generate a sequence of values 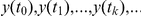. A simple iterative update formula is available,
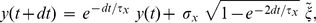
(23)which requires the generation of a Gauss-distributed pseudo-random number 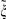 at each iteration, with zero mean and unitary variance [43]. Such an iterative expression is exact, in the sense that 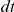 neither needs to be uniform nor infinitesimal for 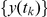 to approximate the statistical properties of 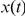 [50]. For very small 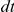 compared to 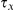, Eq. 23 can be also approximated by a first-order Taylor expansion, leading to
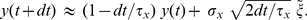
(24)


## Results

In the Materials and Methods section, we have motivated and operatively defined a procedure to derive an effective stochastic version for each ion current composing a conductance-based model neuron. This approximation is entirely based on probability calculus and on analytical expressions derived earlier for experimental channel-noise analysis [6], and it does not require the Fokker-Planck formalism [28], [29]. We have applied here these expressions for synthetic purposes, based on the *a priori* knowledge of the Markov kinetic scheme underlying each voltage- and ligand-gated membrane conductance. The overall conductance associated to each current is modified to include the very same deterministic variables and additive noise term(s), as opposed to previous Langevin-based approaches to *channel noise* macroscopic simulation, where noise terms are (heuristically) applied to the differential equations describing activation and inactivation variables. In addition, the variance and the spectral properties of the extra noise terms are chosen accurately to reproduce the statistical properties of the corresponding microscopic model [6].

In order to assess the validity and accuracy of our approximation procedure, we choose a single-compartmental model neuron and the fast-inactivating and delayed-rectifier sodium and potassium HH currents. We perform Montecarlo microscopic simulations of the exact full Markov model associated to each current, and compare the results to those obtained by its effective macroscopic description. First we test individual ion currents separately as in *voltage-clamp* experiments, upon clamping their gating variable 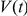, and then we study some passive and active membrane properties, as in *current-clamp* experiments.

### Statistical properties under *voltage-clamp*

**Figure 2:**
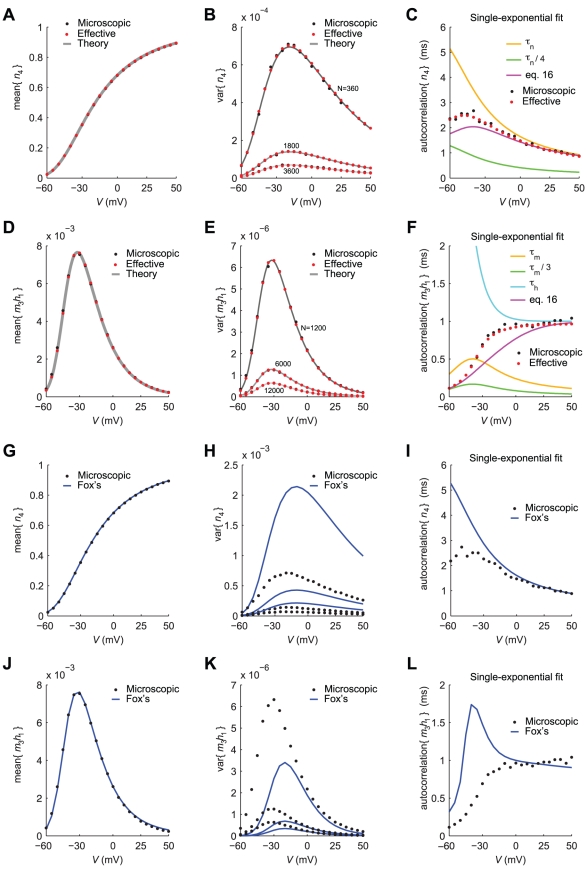
Steady-state statistical properties of the fraction of open channels 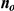, under voltage-clamp. Panels **A–C** refer to delayed-rectifier potassium channels (see Fig. 1B and Table 2), whereas panels **D–F** refer to fast-inactivating sodium channels (see Fig. 1A and Table 2). Black and red dots result from the simulations of the exact kinetic schemes and from our diffusion approximation, respectively. The continuous traces in **A,B,D,E** are drawn by the analytical expressions derived in the text, and refer to an increasing number of simulated channels (namely, 360, 1800, 3600). The dependence on the membrane-patch voltage 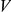 is studied for the mean of 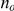 (**A,D**) and for its variance (**B,E**). For an increasing number 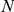 of channels, the variance decreases, as expected. Panels **C,F** show the time constant of the best-fit single-exponential, which approximates the covariance of 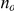 (see Eq. 17). The mismatch between actual best-fit values and the characteristic subunit gating time-constants (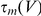, 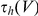, 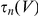, shown for comparison), clearly indicates that great care should be taken in deriving accurate Langevin-kind formulations. Panels **G–L** repeat the very same comparisons presented in panels **A–F**, for the Langevin-approximation introduced by Fox and coworkers (Fox, 1997; Fox and Lu, 1994): the variance of potassium currents is overestimated (**H**), whereas the variance of sodium currents is underestimated (**K**). In addition, the autocorrelation properties are not reproduced correctly (**I,L**).

We keep the membrane voltage 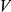 fixed in time, while numerically simulating Eqs. 18, 19. We then study the dependence of the fraction of open channels on 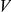 at the steady-state, computing mean, variance and autocorrelation time length of 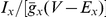, 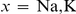. The results confirm that our effective reduction allows one to match accurately the statistical features of the microscopic models, obtained by Montecarlo simulations of the Markov-schemes. Fig. 2 summarises these results for a range of clamped membrane potentials and different total numbers of ion channels. Panels A–C refer to the steady-state properties of HH potassium currents and panels D–F refer to sodium currents. In each panel, black and red markers refer to the actual numerical simulation of the microscopic and the effective models, respectively, whereas solid lines represent the theoretical steady-state values. The mean of the fraction of open channels accurately matches the theoretical predictions (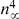 and 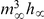 for panels A, D - see Eqs. 13–14) and, as expected, it is independent of the number of channels 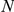. The variance inversely depends on 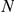 and no difference is evident by comparing microscopic and effective simulations. The solid lines of panels B,E are obtained by plotting 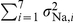 and 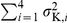 (see Table 2).

For each value of 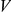, the covariance has a decaying profile characterised by multiple time constants (see Eq. 8 and Table 2). In order to represent concisely how such a decaying profile changes for distinct values of 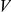, panels C and F show (magenta curves) the values 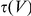 obtained by best fitting with a single exponential 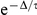 function the autocorrelation function of 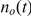. The agreement between microscopic and effective simulations is satisfying and demonstrates that, when predicting and mimicking the autocorrelation properties of channel-noise fluctuations, the kinetic terms 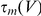, 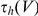, and 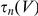, emerging in previous Langevin-based approaches as effective autocorrelation time constants, fail significantly. When a single Ornstein-Uhlenbeck process is used to increase the computational efficiency, the single noise term approximation given in Eqs. 16–17 turns out to be more accurate than the heuristics based on the kinetic time constants 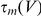, 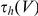, and 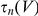 or the submultiples 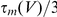, and 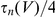 (see also Text S1).

In the lower part of Fig. 2 (panels G–L), the same analysis is repeated, comparing the microscopic Markov-scheme simulations and the results obtained by the Langevin-based approximation proposed by Fox and coworkers [28], [29]. According to the mathematical expressions reported in the Supporting Information, numerical simulations of the Fox's model show that, regardless of the number of channels, the variance of potassium currents is overestimated (panel H), whereas the variance of sodium currents is underestimated (panel K). Because of the inherent limitations of the Langevin-based approach, where a single noise term is added to the differential equations describing activation and inactivation variables, the autocorrelation properties of channel noise fluctuations (panels I,L) are mismatched.

**Figure 3:**
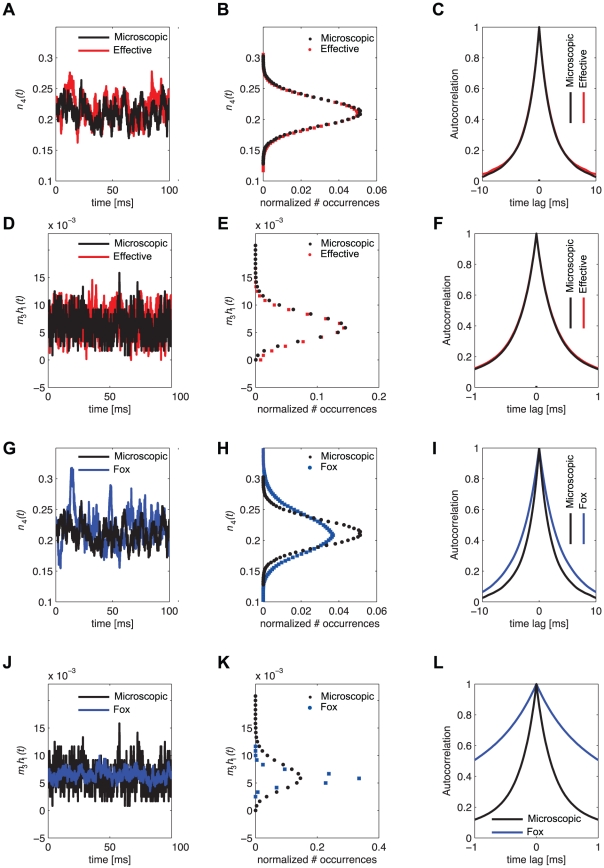
Sample time-series of the fraction of open channels 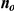, under voltage-clamp (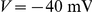). Panels **A–C** refer to delayed-rectifier potassium channels (see Fig. 1B and Table 2), and panels **D–F** to fast-inactivating sodium channels (see Fig. 1A and Table 2). Black and red traces and dots result from the simulations of the exact kinetic schemes and from our diffusion approximation, respectively. The continuous traces in **A,D** are steady-state realisations of the fraction of open potassium and open sodium channels, respectively, while panels **B,E** display the amplitude histogram. Under the conditions considered here (360 potassium and 1200 sodium channels), the Gauss-distributed effective stochastic process approximates well the microscopic model. Panels **C,F** report the autocorrelation function of channel noise fluctuations, demonstrating an excellent agreement of the effective and microscopic simulations (see also Fig. 2C,F). Panels **G–L** repeat the same comparisons presented in panels **A–F**, for the Langevin-approximation introduced by Fox and coworkers (Fox, 1997; Fox and Lu, 1994). As in Fig. 2H,K the variance of potassium currents is overestimated (**G–H**) while the variance of sodium currents is underestimated (**J–K**). In addition, the autocorrelation properties are not reproduced correctly (**I,L**). Additional simulations, for distinct values of the holding membrane potential, are provided as Supporting Information (Figures 5–10 in Text S1).

Finally, Fig. 3 illustrates for 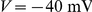 the agreement between the microscopic model and our effective approximation (panels A–F), as well as the mismatch of Fox's algorithm (panels G–L), displaying sample time series of channel noise. Both histograms of fluctuations amplitude (panels B,E,H,K) and autocorrelation functions (panels C,F,I,L) confirm and further support the results of Fig. 2.

### Spontaneous action potential generation

**Figure 4:**
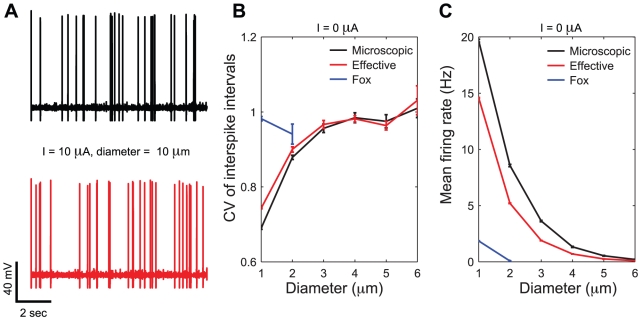
Spontaneous firing in the microscopic and effective models. When weakly depolarising DC currents (**A**, 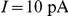) are applied to both the microscopic (black sample trace) and the effective models (red sample trace), the increase in channel noise variances (see Fig. 2C,F) induces a highly irregular spontaneous emission of action potentials, with qualitatively very similar properties. In these simulations, both length and diameter of the neuron are set to 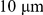, and the single channel conductance for both sodium and potassium channels is 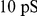. Panels **B,C** show respectively the CV of the ISI distribution and the mean firing rate as a function of cell diameter: results are reported for the microscopic, effective and Fox's models (black, red and blue traces, respectively). The results of panels **B,C** refer to spontaneous activity (i.e., no injected current) with neuron length held fixed at the value 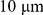.

As the steady-state properties of the fractions of open channels are equivalent in the microscopic and effective models, we tested the full model as in a *current-clamp* experimental protocol. In this case, the gating variable 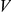 is not clamped to a fixed value and both passive and active membrane properties arise by the interplay between ion currents. Once injected with a weak depolarising DC current 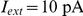, both the microscopic and the effective model neurons fire irregular action potentials [14], as shown in Fig. 4A. In the absence of *channel noise* (i.e., for 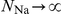 and 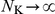), 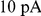 is not strong enough to elicit spiking activity as it is below threshold for (deterministic) excitability.

In order to quantify more accurately this phenomenon, we show in Fig. 4B the coefficient of variation (CV) of the interspike interval distribution obtained simulating the microscopic, effective and Fox's models (black, red and blue traces, respectively), for increasing values of the membrane patch area (i.e., of the number of ion channels). Note that Fox's model exhibits no spontaneous activity for larger cell sizes. On the other hand, the CV of the microscopic and effective models are very close. Fig. 4C shows the corresponding spontaneous mean firing rates: the presence of an “offset” in the results obtained by the effective model is evident, which is greatly reduced as the membrane area increases. This is due to the small number of channels in the membrane patch when the area is very small, against assumption (iii).

### Firing efficacy, latency and jitter in response to monophasic and preconditioned stimuli

**Figure 5:**
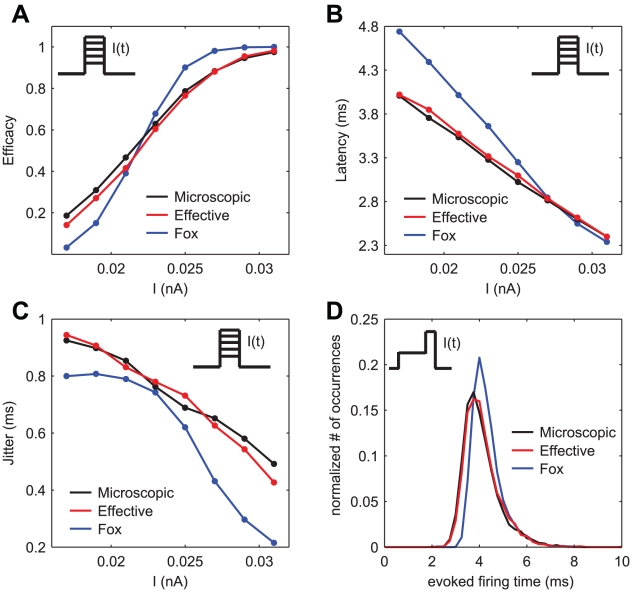
Comparison of firing efficacy, latency and jitter of a sharp current pulse. Panels **A**, **B** and **C** display the firing efficacy, the average latency and the jitter of the evoked responses, respectively, after the application of a monophasic stimulus of duration 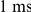 repeated for 10000 trials. Black and red traces and dots result from the simulations of the exact kinetic schemes and from our diffusion approximation, and in blue we indicate the results from the simulation of the Langevin-approximation introduced by Fox. Panel **D** shows the distribution of spike occurrence times, evoked by a biphasic stimulus over 10000 trials: the duration and amplitude of the preconditioning part are 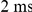 and 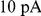, respectively, the duration and amplitude of the second part are 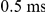 and 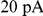. In all panels, the neuron is simulated as a single cylindrical compartment of length and diameter equal to 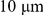 and single channel conductances equal to 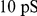, for both sodium and potassium channels. The integration time step was set to 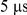.

In order to perform a direct comparison with the analysis carried out in [27], a monophasic current pulse of fixed duration and increasing amplitude was applied 10000 times to probe the impact of channel noise on neuronal evoked responses. In Fig. 5, panel A displays the firing efficacy (i.e., the fraction of trials where a spike was elicited), panel B shows the average latency of the evoked action potential with respect to the stimulation time, and panel C displays the standard-deviation (i.e., the jitter) of the firing latency. Black and red traces and dots result from the simulations of the exact kinetic schemes and from our diffusion approximation, respectively, while in blue we indicate the results from the simulation of the Langevin-approximation introduced by Fox. The satisfactory agreement between microscopic and effective models is apparent, whereas simulations according to Fox's algorithm differ considerably. Panel D shows the distribution of spike occurrence times, evoked by a biphasic stimulus over 10000 trials. The distributions of spike times obtained by the microscopic and effective models almost overlap, while Fox's distribution has a significantly different shape.

The results we present here for the microscopic and Fox's models are in close agreement with those discussed in greater detail in [27].

### Reliability of evoked spike timing and response latency

**Figure 6:**
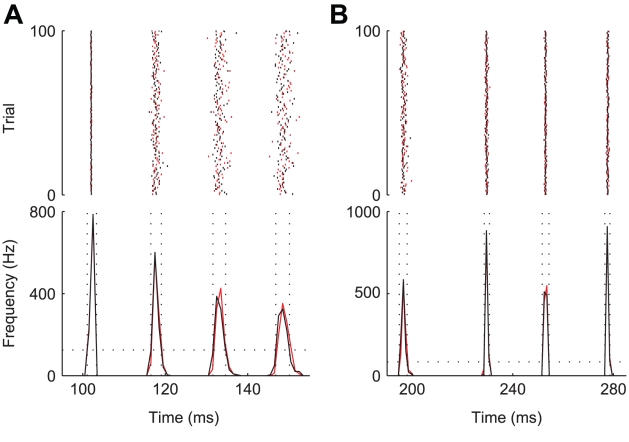
Raster plots and peristimulus time histograms (PSTH) for the timing of spiking responses to repeated identical DC pulses (A) and fluctuating currents (B). Red traces and markers refer to Montecarlo microscopic simulations of the full model, while black traces and markers refer to the effective model. The values of reliability (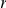) and precision (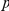) are in accordance with those measured in *in vitro* experiments. In particular, in panel A: 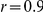, 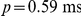 for the microscopic model, 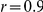, 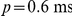 for the effective model. Panel B: 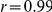, 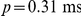 for the microscopic model, 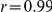, 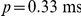 for the effective model. The DC pulse has an amplitude of 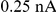, whereas the noisy stimulus is the same realisation of an Ornstein-Uhlenbeck's process, with mean and standard deviation set to 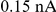, and with autocorrelation time length set to 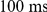.

The results shown in Fig. 5 refer to the application of either a mono- or biphasic stimulus of *short* duration, in the order of milliseconds. Here, we extend the previous analysis to the case of significantly longer stimulations: our objective is to study the so-called *reliability* of spike timing along the lines of the experimental protocol defined in [13]. It is well known that, as a consequence of *channel noise*, the reliability of evoked spike timing is higher for current stimuli 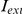 fluctuating in time than for DC current pulses [13], [15], [17]. Indeed, larger fluctuations induced in the membrane potential by the driving stimulus transiently hyperpolarise the cell, thus reducing the variance of *channel noise* (see Fig. 2B,E). A similar phenomenon has been described in the case of inhibitory autapses in the cerebral cortex [53] and it could also be represented at microcircuit-level by the role of disynaptic inhibition [54]. A single-compartmental model simulation incorporating *channel noise* can replicate this effect [15] and constitutes a further benchmark to compare microscopic and effective models. We note that for this analysis, we have chosen the neuron parameters in order to reproduce the results presented in [13]. The agreement between models is very good as shown in Fig. 6, where black (red) traces and markers refer to the microscopic (effective) model. The spike responses to two repeated identical stimuli were considered: a DC pulse (panel A) and a realisation of an exponentially-filtered white noise (panel B). The raster diagrams of the spike times (upper plots), as well as the corresponding time histograms (lower plots), demonstrate that the two models perform in the same way as the spread and latency of the spike times, in response to the repeated identical stimulation, are practically identical. Finally, a quantitative measure of both precision and reliability (computed according to [13]) provides values similar to those measured in *in vitro* experiments (see figure caption).

### Frequency-current (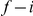) response curves

**Figure 7:**
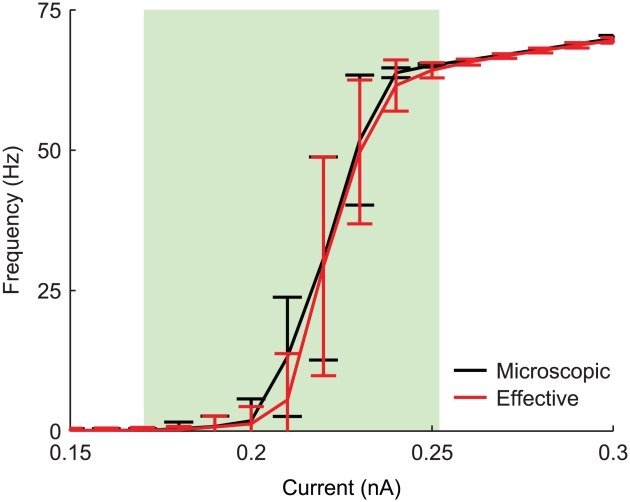
Frequency-current (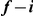) response curves. Mean firing rate, in response to a DC current injection, studied for increasing stimulus intensities in both Montecarlo microscopic (black trace) and effective model (red trace) simulations. Single-channel conductance for both sodium and potassium channels set to 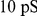.

For stronger depolarising DC currents 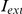, the firing of both the microscopic and the effective models becomes more regular. The mean firing rate, as a function of 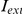 was studied to test the agreement between their evoked response properties. Fig. 7 shows the 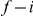 curves computed over 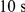-long evoked spike-trains. For each current amplitude, the simulation was repeated 10 times, and firing rates obtained in each repetition were averaged. Error bars indicate the standard deviation of the firing rate across repetitions. Responses of both the microscopic and the effective models result in almost identical variability across repetitions and in both cases the type-II behaviour, typical of the deterministic HH model, fades away. This is a known consequence of the presence of *channel noise*, which smooths what would be an abrupt transition from a quiescent to a spiking regime. These irregular transitions occur for both models in the very same range of input currents (green-shaded region in the figure), where the membrane potential repeatedly switches between a resting equilibrium point and a spiking limit cycle (see [15] for an extended discussion).

### Power-spectral density of membrane voltage fluctuations

**Figure 8:**
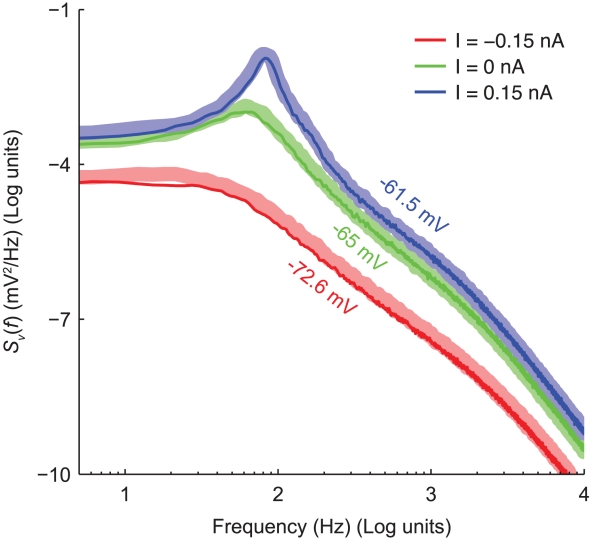
Voltage power spectral densities of subthreshold membrane potential trajectories. Comparison between the microscopic (thick shaded lines) and the effective (thin solid lines) models. 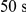 of simulated recordings of the membrane potential were obtained under weak holding currents ({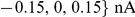), resulting in membrane potential traces fluctuating around an offset ({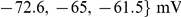). Rare spontaneous spikes were removed from the analysis, excluding the 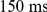 preceding and the 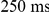 following each spike. The spectra have been obtained by applying the Welch method, on moving windows of duration 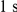 and overlapping by 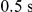, and subsequently averaging the results.

We finally compare the power-spectral densities of subthreshold membrane potential trajectories, obtained in simulations of the microscopic and effective models. We followed closely the numerical analysis of [18], where a comparison between the microscopic model and a quasi-active linearised model with phenomenological inductances was instead presented. Once more, the agreement between the two models is satisfactory: in Fig. 8 we show the results, indicating by thick shaded curves the power spectra computed from the microscopic model, and by thin solid lines the power spectra computed from the effective model. The agreement is good over the entire frequency domain, reproducing some of the features that have been experimentally measured in cortical neurons and related to *channel noise* [19].

## Discussion

In this paper, we introduced the systematic generalisation and improvement of previous Langevin-based channel-noise effective simulation techniques. By the diffusion approximation of ion channels population dynamics, we aimed at efficient and accurate computer simulation of *channel noise*. Our method approximates correctly the statistical properties of individual ion conductances (their mean and autocovariance function), matching those emerging from the Montecarlo simulation of their corresponding Markov schemes. In addition, both passive and active properties of neuron model simulations are replicated with satisfying accuracy. While simulating 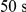 of model time by a conventional Montecarlo algorithm takes about 22 hours for completion, the same simulation with very similar statistical features is replicated by the effective model in only 124 seconds, on a machine equipped with a 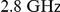 Intel Core i7, with 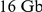 of RAM, running Ubuntu Linux 9.10. When relating the computation times to the benchmarking provided by [27], our diffusion approximation is only 1.5 times slower than Fox's algorithm and therefore more than 4.5 times faster than the fastest available algorithm for exact microscopic simulations [14]. Our results have been obtained by custom C++ and NEURON model simulations (see the Materials and Methods section), but the implementation of the method in other languages (MATLAB, Python) or other simulation environments (Genesis, NEST, Brian) is straightforward. Besides the speed increase, the value of our contribution is threefold: i) mean, variance and spectral properties of fluctuations induced by the stochasticity of individual ion currents are correctly approximated, regardless of the number of channels; ii) our method is presented operatively, allowing any deterministic neuron model, whose ion conductance kinetics is described by a Markov scheme, to be quickly converted into an equivalent stochastic version without involving any heuristics on the choice of the parameters for extra noise sources; iii) the underlying assumptions for the validity of our approximation are also indicated with full details.

The earlier proposals of [28], [29], recently challenged for their accuracy, are indeed very similar to our method, although focused only on the HH model. In these papers, the equations that state variables 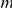, 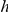, and 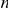 obey to are modified by adding a single noise term 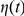, as follows:
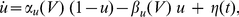
(25)where 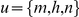 and 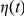 is a Gauss-distributed noise term with zero mean and covariance given by
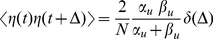
(26)


By direct inspection and comparison of Eqs. 4, 5, 6, and 20, it is possible to show that Eq. 25 and Eqs. 4–5 are equivalent (see Text S1). In other words, for 2-state kinetics the approximation given by Eq. 25 is correct but fails when the powers 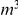, 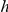, and 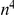 are computed and when they are combined in the product 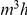. Under these circumstances, mean, variance and covariance function indeed deviate considerably from the correct dependence on 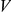, emerging from the microscopic simulations or computed analytically (see Text S1). Briefly, the potassium current simulated by the fourth power 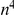 overestimates the correct variance, does not share the correct mean and has qualitatively different autocorrelation properties. The sodium current simulated by the third power 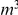 and the product by 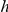 instead underestimates the correct variance, does not share the correct mean and has quantitatively different autocorrelation properties. The interested reader can find all the details in the Supporting Information. We believe that the reason for the success of our approximation, compared to Fox's approach, lies not only in the correct agreement of fluctuations mean and variance, validated by direct comparison with the theoretical and numerical results of the *microscopic* description [31], but also in the fact that the covariance function of those fluctuations must be precisely matched and should be approximated by a sum of white-noise terms and not by adding noisy terms to the deterministic kinetic equations for activation and inactivation variables. However, we note that under current-clamp condition, there is no *a priori* guarantee that any Langevin-based approach, including our diffusion approximation, works faithfully [55]. In fact, our assumption (v), that the gating variable (e.g., 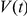) changes slowly compared to channel kinetics, may not be *instantaneously* satisfied during very fast transients. Although the same condition is anyway employed for obtaining numerical speed-up in deterministic conductance-based models [1], [56], [57], the instantaneous channel noise fluctuations might lag behind what predicted by microscopic exact models (see Figs. 11–12 in Text S1). Nevertheless, owing to the satisfying results we obtained in terms of firing-rate properties, firing time reliability, precision, efficacy, latency, jitter as well as subthreshold membrane fluctuations, we speculate that inaccuracies during very fast transients might still be compatible with accurate model performances (perhaps due to the low-pass properties of the membrane), provided that first- and second-order voltage-clamp statistics are correctly matched.

A very similar reduction procedure is implicitly mentioned in [18], where the authors developed a quasi-active membrane potential equation employed only for the spectral analysis of subthreshold voltage noise, but not for its actual numerical simulations. The authors state clearly that their approximation can be viewed as a linearised approximation of the Fokker-Planck master equation [29]. As opposed to our method, which requires adding multiplicative noise terms to the membrane potential equation, their quasi-active model includes only additive noise, upon linearisation, resulting in the definition of electrical circuit analogs (capacitances and inductors) useful for the intuitive understanding of *channel noise* for subthreshold passive membrane properties, and for the analytical prediction of the spectral properties of membrane potential fluctuations. The authors, however, do not explicitly provide any derivation of their approach and do not test it for the excitable response neuronal properties as a replacement of microscopic simulations.

One further approach to *channel noise* modelling has been proposed in [35]. We share the motivation of performing accurate and fast simulation by a Langevin-based approach, but we use stochastic processes with precise and defined statistical properties, coincident with those emerging from the microscopic description of the stochastic behaviour of channels. In the proposal by [35], the effective stochastic term is modelled as Brownian motion, i.e., as a Gauss-distributed process with independent increments and heuristically fixed constant variance, ignoring its voltage-dependence and the variety of autocorrelation time constants. Since the analytical derivation of the accurate statistical properties of *channel noise* is possible, and its implementation straightforward as we showed here, there is no need to use arbitrary parameters for simulating the stochastic components of ion currents gating.

It is worth mentioning that *population density* approaches, proposed for integrate-and-fire as well as conductance-based models [58]–[61], share to some extent the motivations of our work: exploring the impact of endogenous or exogenous noise sources while developing tools to capture or effectively simulate population-level dynamics [62], [63]. Those works also aim at correctly mimicking actual network interactions in terms of an equivalent stochastic additive input to a generic unit of the network [64], as in the *mean-field* approximation of synaptic interactions [65]. Since our work provides an accurate effective description of an intrinsic (multiplicative) noise source, our formulation could be very relevant for those approaches, in extending population density descriptions to incorporate endogenous channel noise.

In conclusion, we believe that our method could open new possibilities for the investigations of *channel noise* impact in morphologically detailed conductance-based model neurons, as well as in large networks models, where realism cannot be compromised by computational parsimony. Spike timing computation in neural networks [66] with specific microcircuit architectures [54] might be for instance easily complemented by stochastic components of neural excitability, employing detailed neuron models. Finally, the possibility of further increasing the level of approximation, involving only a modification of the spectral properties of *channel noise* without affecting the accuracy of its variance, may lead to an in depth understanding of what temporal correlation properties are relevant for specific computational neuronal properties and how *channel noise* interacts with other noise sources.

## Supporting Information

Text S1 This supporting information reviews a few results of the theory of stochastic processes, useful for supporting our discussion and for the comparison between Fox's and our method. It also contains Figures where extended numerical comparisons between Fox's and our method are presented.
(2.06 MB PDF)
