# Supplementary material for: Accurate and Fast Simulation of Channel Noise in Conductance-Based Model Neurons by Diffusion Approximation
Source: PLoS Comput Biol. 2011 Mar 10;7(3):e1001102. doi: 10.1371/journal.pcbi.1001102 (PMC3053314; doi:10.1371/journal.pcbi.1001102)
Supplement: Text S1 — This supporting information reviews a few results of the theory of stochastic processes, useful for supporting our discussion and for the comparison between Fox's and our method. It also contains Figures where extended numerical comparisons between Fox's and our method are presented. (2.06 MB PDF) [file pcbi.1001102.s001.pdf]

Supporting Information: Text 1

In this document we review some results from the theory of stochastic processes, useful for supporting our discussion on similarities and differences between Fox's and our method. We start by verifying the accuracy of the standard numerical method, employed for generating realisations of an Ornstein-Uhlenbeck process. We then compare Fox's equation(s) to an Ornstein-Uhlenbeck process to help us derive its steady-state statistical properties and we point out its inaccuracies, when employed for approximating the exact simulation of a Markov kinetic scheme.
The code for generating each of the panels of Figures 1-4 is available on ModelDB (see the main text), as script in MATLAB (Mathworks, Natick, USA).

Consistent numerical simulation of stochastic differential equations

We consider the continuous-time version of a stochastic differential equation, which defines the Ornstein-Uhlenbeck process *x(t)*. We may therefore write:

$$\tau_x \dot{x} = -x + \sigma_x \sqrt{2\tau_x}\xi(t) \qquad (1)$$

In Eq. 1, *ξ(t)* is a Gauss-distributed continuous-time process, characterized by zero mean and covariance given by a Dirac's delta function *δ(Δ)*. By stochastic process theory and probability calculus (Cox and Miller, 1965; Papoulis and Pillail, 2002), we know that *x(t)* is a non-stationary stochastic process with mean and covariance given by the following expressions, where x_0 is the initial condition of *x(t)*:

$$< x(t) > = \; x_0 \; e^{-(t-t_0)/\tau_x} \qquad (2)$$

$$\begin{aligned} &< (x(t+\Delta) - \bar{x}(t+\Delta))\,(x(t) - \bar{x}(t)) > = \\ &\quad = \; {\sigma_x}^2 \; \left(1 \; - \; e^{-2\;(t-t_0)/\tau_x}\right) \; e^{-|\Delta|/\tau_x} \end{aligned} \qquad (3)$$

By means of a standard iterative numerical simulation procedure, already reviewed in the main text, a realisation of the discrete-time process $y_k = y(t_k) = y(k\,dt)$, k=1,2,3,... can be generated, to equivalently approximate *x(t)*:

$$y(t+dt) \;\approx\; (1 - dt/\tau_x)\; y(t) + \; \sigma_x \; \sqrt{2dt/\tau_x} \; \tilde{\xi} \qquad (4)$$

The noise term in Eq. 4 is generated as a pseudo-random number with normal distribution by the Box-Muller algorithm (Press *et al.*, 2007) (for instance, by using the MATLAB `randn` command). The accuracy of the equivalence $x \sim y$ is confirmed by the results shown in Figure 1.

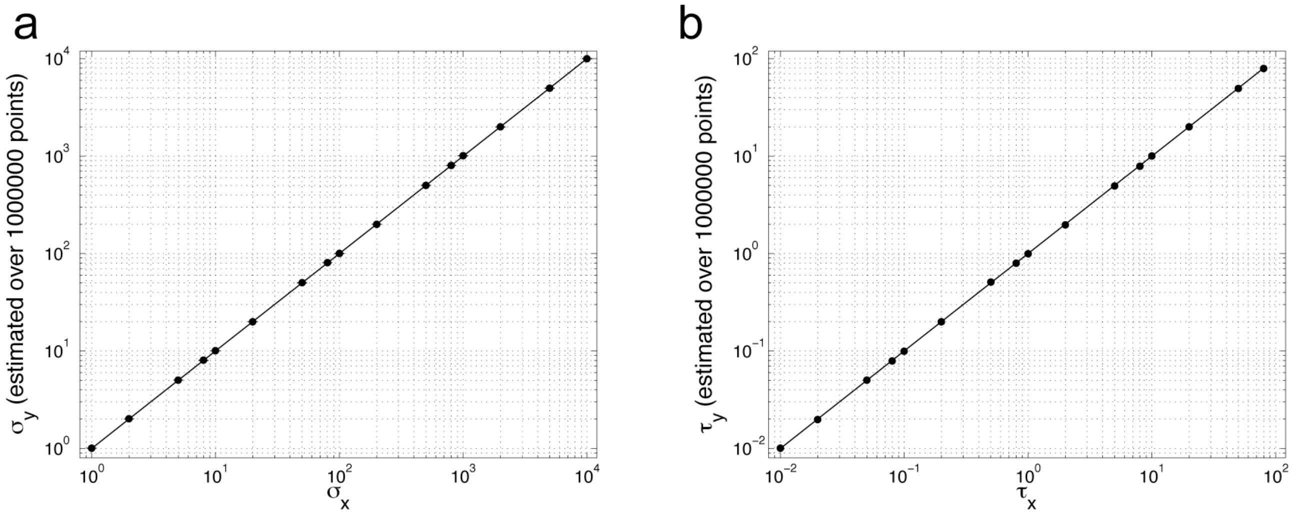

Figure 1: The statistical properties of the continuous-time Ornstein-Uhlenbeck stochastic process *x(t)* and of its discrete-time approximation y_k are in agreement: the covariance function is captured both in its peak amplitude (the variance of the process) and in its shape (a mono-exponential decay). Markers represent (**a**) the standard-deviation of y_k, estimated by the MATLAB `std` command, and (**b**) the autocorrelation time-length of y_k, estimated by the `xcov` and `fit` commands, obtained from one million values of y_k. Each parameter combination was repeated 5 times and the standard-deviation of each estimate is represented as an error bar, which is smaller than the marker size. The continuous line is the unitary slope line and is plotted to indicate ideal agreement. Parameters employed for the simulations: (**a**) $\tau_x = 1$ ms, (**b**) $\sigma_x = 50$; dt was set to $\tau_x/80$.

The definition of the stochastic process as introduced by Fox and collaborators (Fox and Lu, 1994; Fox, 1997) consists in adding a noise term to the right-hand side of each kinetic equation, associated for example to the variables *m*, *h*, and *n* of the Hodgkin-Huxley model. In continuous-time, and abusing the notation, for a generic variable *u* the following equation holds:

$$\frac{du}{dt} = \alpha_u(V)\ (1-u) - \beta_u(V)\ u\ +\ \eta(t) \qquad (5)$$

For our next discussion, as well as for Figs. 2-3, the coefficients α_u and β_u are considered constant, under the explicit hypothesis of clamping the value of the membrane potential *V*. The term *η(t)* is a realization of a Gauss-distributed continuous-time process, characterized by zero mean and delta-correlated covariance as in the Ornstein-Uhlenbeck process:

$$< \eta(t)\eta(t+\Delta) >= \frac{2}{N}\frac{\alpha_u\ \beta_u}{\alpha_u+\beta_u}\delta(\Delta) \qquad (6)$$

By setting $\tau_x = 1 / (\alpha_u + \beta_u)$, $u_\infty = \alpha_u / (\alpha_u + \beta_u)$, $\sigma_x{}^2 = N^{-1}\ \alpha_u\ \beta_u / (\alpha_u + \beta_u)^2$, and $x = u - u_\infty$, Eqs. 5 and 1 coincide. Then, by analogy and direct inspection, we adapt the considerations about *x(t)* to *u(t),* stating that *u(t)* is a Gauss-distributed non-stationary process, with mean and covariance given by the following expressions, where u_0 is the initial condition of *u(t)*:

$$< u(t) > =\ (u_0 - u_\infty)\ e^{-(t-t_0)/\tau_x}\ +\ u_\infty \qquad (7)$$

$$< (u(t+\Delta) - \bar{u}(t+\Delta))\,(u(t) - \bar{u}(t)) > = \\ = \sigma_x{}^2 \left(1 - e^{-2\,(t-t_0)/\tau_x}\right) e^{-|\Delta|/\tau_x} \quad (8)$$

Along the same line of reasoning, we anticipate the validity of the numerical method to generate a discrete-time process $y_k = y(t_k) = y(k\,dt)$, k=1,2,3,..., equivalent to $u(t)$:

$$y(t + dt) \approx [1 - dt\,(\alpha_u + \beta_u)]\; y(t) + dt\,\alpha_u + \sqrt{2dtN^{-1}\,\alpha_u\beta_u/(\alpha_u + \beta_u)}\;\bar{\xi} \quad (9)$$

More explicitly, y_k and $u(t)$ share the same steady-state mean ($\alpha_u / (\alpha_u + \beta_u)$), the same variance ($N^{-1}\alpha_u\,\beta_u/(\alpha_u + \beta_u)^2$), and an exponentially decaying covariance function with time-constant $1 / (\alpha_u + \beta_u)$. We verified these considerations numerically, as summarized by the results in Figure 2.

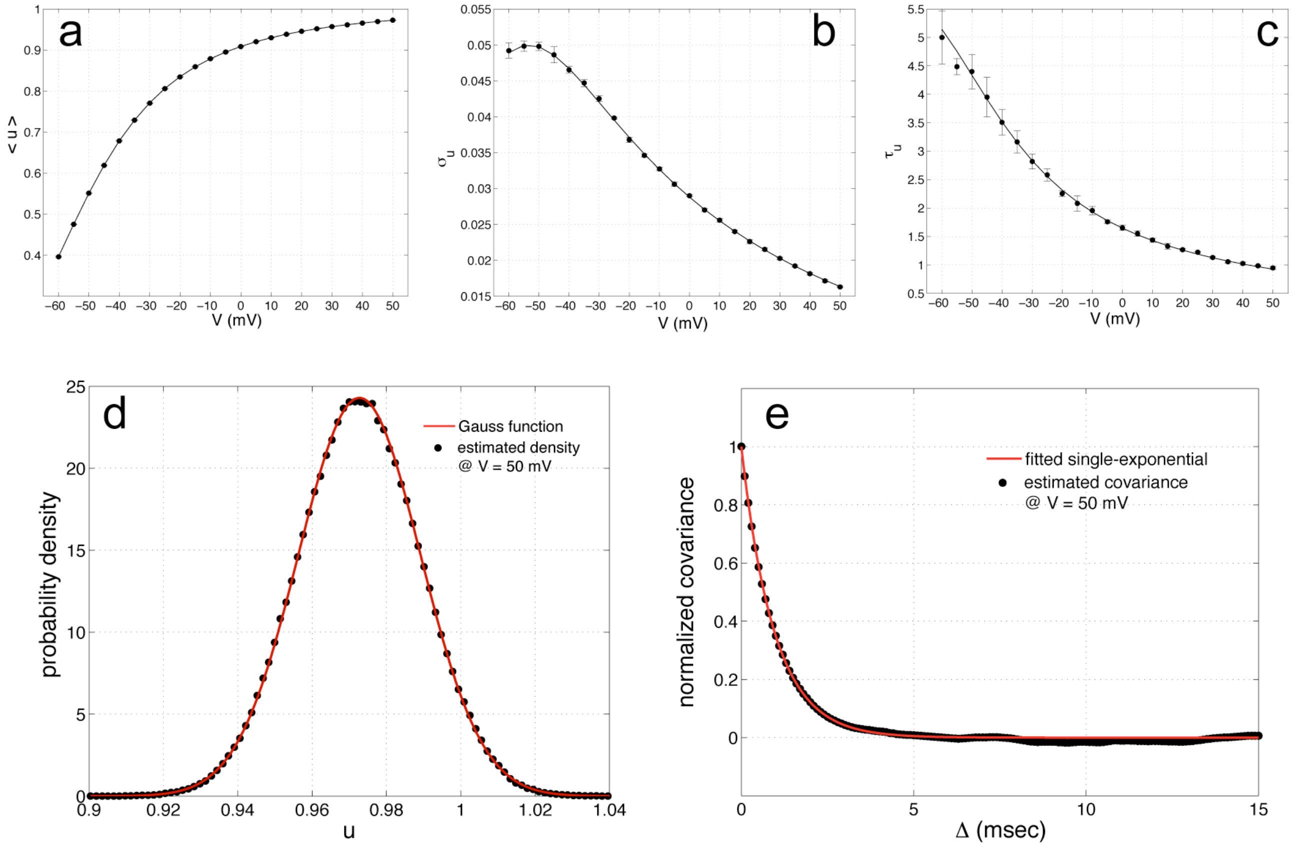

Figure 2: Comparison between the predicted (continuous black lines) and the actual statistical properties (markers) of the discrete-time process y_k that approximates $u(t)$. Markers represent (**a**) the actual mean, estimated by the MATLAB `mean` command, (**b**) the standard-deviation, estimated by the MATLAB `std` command, and (**c**) the autocorrelation time-length, estimated by the `xcov` and `fit` commands, obtained from ten million values of y_k. Each parameter combination was repeated 5 times and the standard deviation of each estimate is represented as an error bar, mostly smaller than the marker size. Panels (**d-e**) confirm that the distribution of y_k, estimated by the MATLAB `hist` command, is gaussian and that its (normalised) covariance, estimated by the MATLAB command `xcov`, is a decaying single-exponential function, with the expected time constant. Parameters employed for the simulations: dt = 0.001 ms and N = 100, while α_u and β_u were chosen as those of the Hodgkin-Huxley potassium current.

Preliminaries on the powers of a Gauss-distributed stochastic process

When x is a generic continuous-valued Gauss-distributed random variable, with mean μ and variance σ^2, its probability density is by definition

$$f_x(X) \; = \; \frac{1}{\sqrt{2\pi\sigma^2}} \; e^{-\frac{(X-\mu)^2}{2\sigma^2}} \tag{10}$$

The powers of x ($y = x^i$ with i>0), are no longer Gauss-distributed. For instance, when $i = 2$, x^2 is distributed according to a non-central chi-square distribution, with a single degree of freedom (Papoulis and Pillail, 2002).
In the general case ($i > 0$), the probability density function reads (Klugman *et al.*, 2008)

$$f_y(Y) \; = \; Y^{\frac{1-i}{i}} \; \frac{1}{i \; \sqrt{2\pi\sigma^2}} \; e^{-\frac{(Y^{1/i}-\mu)^2}{2\sigma^2}} \tag{11}$$

However, as x is Gauss-distributed, calculating mean and variance of y for integer n involves only algebraic derivations, requiring the raw moments of x, $<x^i>$ where i = 1,2,3,.... These are available, as polynomials in μ and σ^2 (Papoulis and Pillail, 2002). We provide some of these derivations (for i = *2, 3,* and *4*), indicating by μ_i, σ_i^2 mean and variance of x^i:

$$\mu_2 \; = \; \mu^2 \; + \; \sigma^2 \qquad\qquad \sigma_2^2 \; = \; 2\sigma^2 \; (\sigma^2 \; + \; 2\mu^2) \tag{12}$$

$$\mu_3 \; = \; \mu \; (\mu^2 \; + 3\sigma^2) \qquad\qquad \sigma_3^2 \; = \; 3\sigma^2 \; (3\mu^4 + 12\mu^2\sigma^2 + 5\sigma^4) \tag{13}$$

$$\mu_4 \; = \; \mu^4 \; + \; 6\mu^2\sigma^2 \; + \; 3\sigma^4 \qquad\qquad \sigma_4^2 \; = \; \sigma^2 \; (16\mu^6 \; + \; 168\mu^4\sigma^2 \; + \; 384\mu^2\sigma^4 \; + \; 97\sigma^6) \tag{14}$$

Finally, if the covariance of the original process *x(t)* is a decaying single-exponential function with time constant τ_x (as in an Ornstein-Uhlenbeck process) the covariance of its integer powers $x^i(t)$ takes the form of a linear combination of decaying exponentials, each weighted by polynomials in μ and σ^2. We don't include here the derivation but we note that for $i = 2$, there is only one exponential term in the covariance, weighted by σ_2^2, with time constant τ_x / 2. For $i = 3$, there are three exponentials, with time constants τ_x / 3, τ_x / 2, and τ_x. For $i = 4$, there are two exponentials, with time constants τ_x / 4, and τ_x / 2.

Steady-state statistics of the term n^4

We can now derive analytically the statistical properties of the integer powers of Fox's stochastic process *u(t)*. In this section, we consider u = n, representing the kinetic variable associated to Hodgkin-Huxley potassium currents. Figure 3 shows the results of numerical simulations for $n^4 = u^4$, under the very same conditions employed in Figure 2. These results confirm that mean and variance of u^4 are correctly accounted for by Eqs. 14, which have been used to draw the black continuous curves of Figure 3.

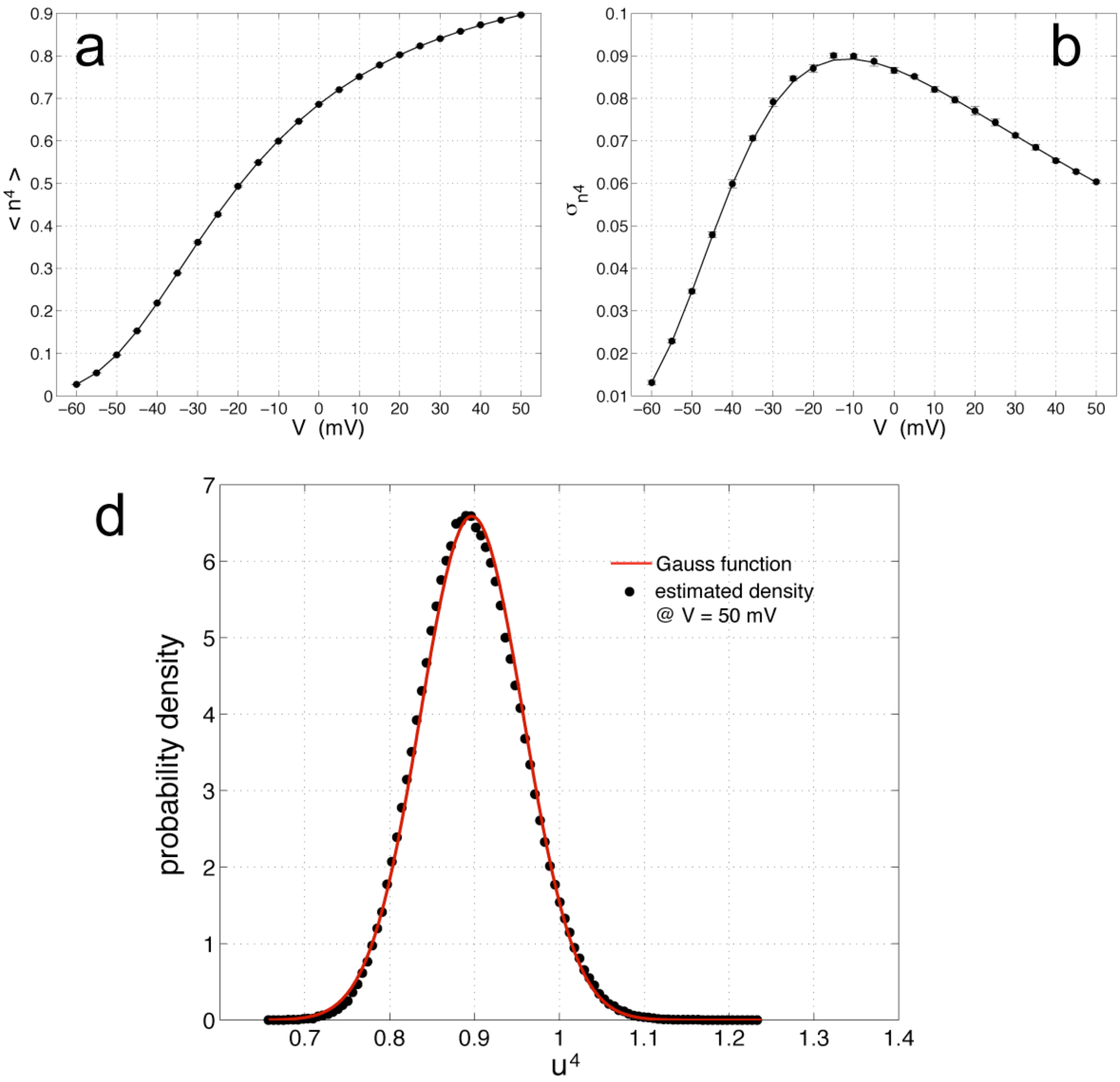

Figure 3: Comparison between the predicted (continuous black lines) and the actual (markers) statistical properties of the discrete-time process $(y_k)^4$ that approximates $n^4(t) = u^4(t)$. Markers represent (**a**) the actual mean, estimated by the MATLAB `mean` command, and (**b**) the standard-deviation, estimated by the MATLAB `std` command, obtained from ten million values of y_k. Each parameter combination was repeated 5 times and the standard-deviation of each estimate is represented as an error bar, mostly smaller than the marker size. Panel (**d**) confirms the expected deviation from a Gauss distribution for the values of $(y_k)^4$, estimated by the MATLAB `hist` command. Parameters employed for the simulations: dt = 0.001 ms and N = 100, whereas α_u and β_u were chosen as those of the Hodgkin-Huxley potassium current.

The knowledge of these analytical expressions, as well as their numerical verification, is particularly important as the fourth power of *n(t) = u(t)* is proposed to macroscopically approximate the statistics of the open fraction of a population of N delayed-rectifier channels. However, in the main text of the paper we have shown how to compute these statistics, starting by the theory of stochastic channel opening and employing those results for our method (see Table 2 in the main text). We can therefore check whether or not these statistics are correctly captured by those of n^4. The answer is negative, as shown in Figure 4,a-b. Indeed, the mean of the fraction of open potassium channels (microscopic exact description) is given at the steady-state by (see Eqs. 13, 18)

$$\frac{\alpha^4}{(\alpha+\beta)^4} \qquad (15)$$

The mean of n^4 is not identical to the above expression, but converges to it only for large N (~100 and more, see Fig. 4a), differently from our method, which is in agreement for any N. For finite N, Fox's process n^4 slightly over-estimates the true mean as its expression reads

$$\frac{\alpha^4}{(\alpha+\beta)^4} + \frac{3\ \alpha^2\ \beta\ (2\ N\ \alpha\ +\ \beta)}{N^2\ (\alpha\ +\ \beta)^4} \tag{16}$$

The variance of the fraction of open potassium channels (microscopic exact description) is given at the steady state by the sum of 4 variance terms (see Table 2)

$$\frac{\alpha\ \beta}{N\ (\alpha\ +\ \beta)^8}\ \alpha^3\ (4\ \alpha^3\ +\ 6\ \alpha^2\ \beta\ +\ 4\ \alpha\ \beta^2\ +\ \beta^3) \tag{17}$$

The variance of n^4 over-estimates the correct values (see Fig. 4b), as it reads (from Eq. 14)

$$\frac{\alpha\beta}{N(\alpha+\beta)^8}\alpha^3\left(16\alpha^3+\frac{168}{N}\alpha^2\beta+\frac{384}{N^2}\alpha\beta^2+\frac{97}{N^3}\beta^3\right) \tag{18}$$

In addition, the covariance of n^4 contains only two exponential terms, with time constants τ_n / 4 and τ_n / 2, while the true covariance of the process should contain four terms (see Eqs. 9-10,15), with time constants τ_n / 4, τ_n / 3, τ_n / 2, and τ_n (see Table 2).

Steady-state statistics of *m^3h*

We can also derive analytically the statistical properties of two independent Fox's stochastic processes *m(t)* and *h(t)*, generated by employing α_m, β_m *and* α_h, β_h in Eq. 5, respectively. In particular, we want to calculate mean and variance of their product, after cubic power of *m(t)*. Because of the statistical independence, the mean of the product $m^3(t)h(t)$ is the product of the means $\mu_{3,m}$ μ_h, while variance is given by the expression $\sigma_{3,m}^2\ \sigma_h^2 + \sigma_{3,m}^2\ \mu_{1,h}^2 + \sigma_h^2\ \mu_{3,m}^2$ (refer to Eqs. 12-13; Papoulis and Pillail, 2002).
As for potassium currents, the mean of $m^3(t)h(t)$ converges to the true value

$$\frac{\alpha_m^3}{(\alpha_m+\beta_m)^3}\frac{\alpha_h}{(\alpha_h+\beta_h)} \tag{19}$$

for large N (~1000 and more, see Fig. 4c). The variance of $m^3(t)h(t)$ however under-estimates the correct values (see Fig. 4c).
In addition, because of the statistical independence, the covariance of the product is the product of the covariances (see Eq. 15; Conti & Wanke, 1975) so that the covariance of $m^3(t)h(t)$ can be calculated. It contains the correct number (seven) of exponential terms, however each weighted by the wrong variance coefficient (see Eqs. 9-10,15 and Table 2).

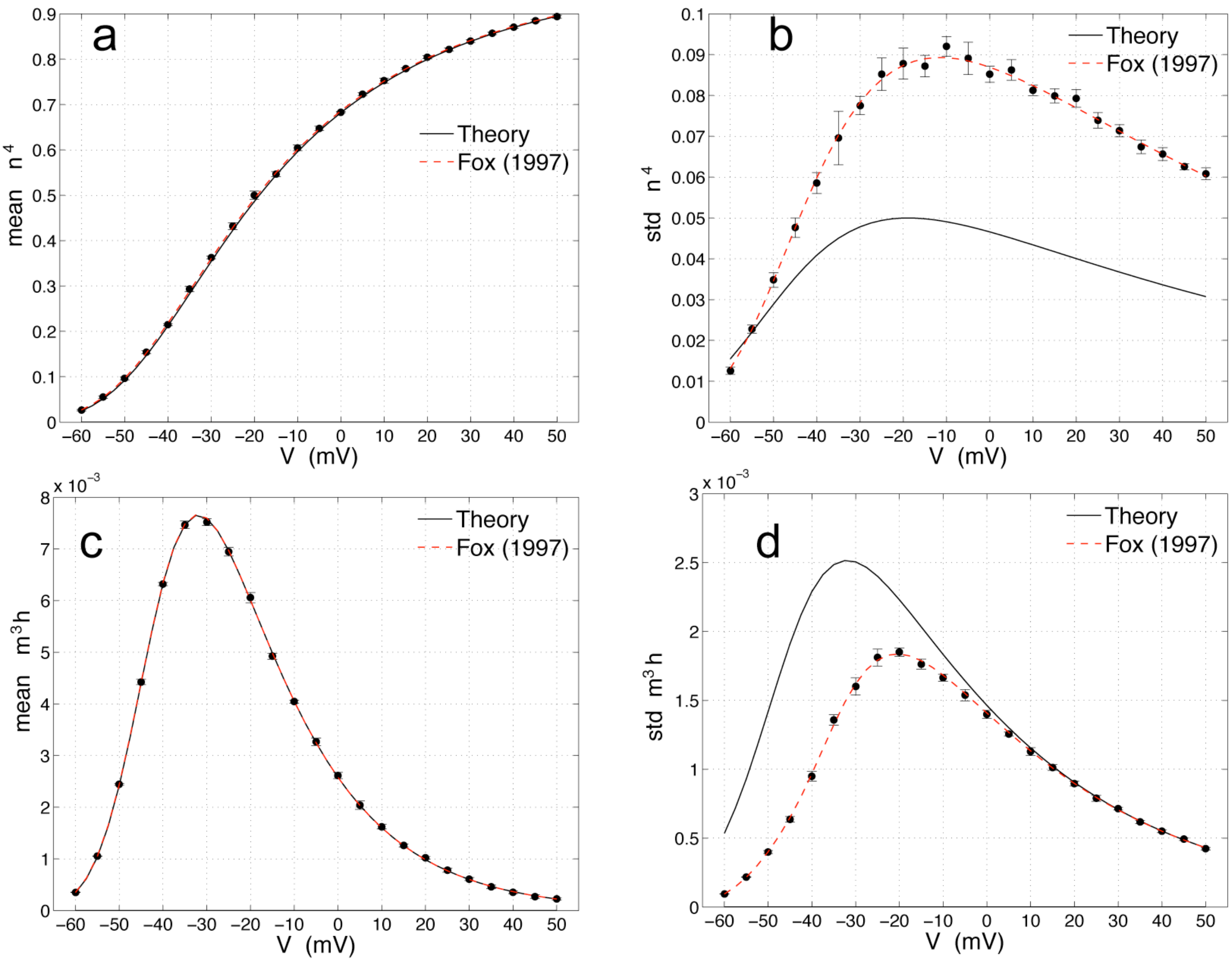

Figure 4: Comparison between the predicted (continuous lines) and the actual statistical properties (markers) of (**a-b**) $n^4(t)$ and (**c-d**) $m^3(t)h(t)$. Markers represent the actual numerical simulations of the Fox's stochastic process(es), estimating (**a**) the actual mean, by the MATLAB `mean` command, and (**b**) the actual standard deviation, by the MATLAB `std` command, obtained from one million values. The red dashed line is drawn according to the formulae derived in this supplemental material. The black continuous lines represent the mean and standard deviation calculated by the theory and coincident with those employed by our method. Each parameter combination was repeated 5 times and the standard deviation of each estimate is represented as an error bar, mostly smaller than the marker size. Parameters employed for the simulations: dt = 0.001 ms and N_K = 100, N_{Na} = 1200.

Additional Supplemental Figures

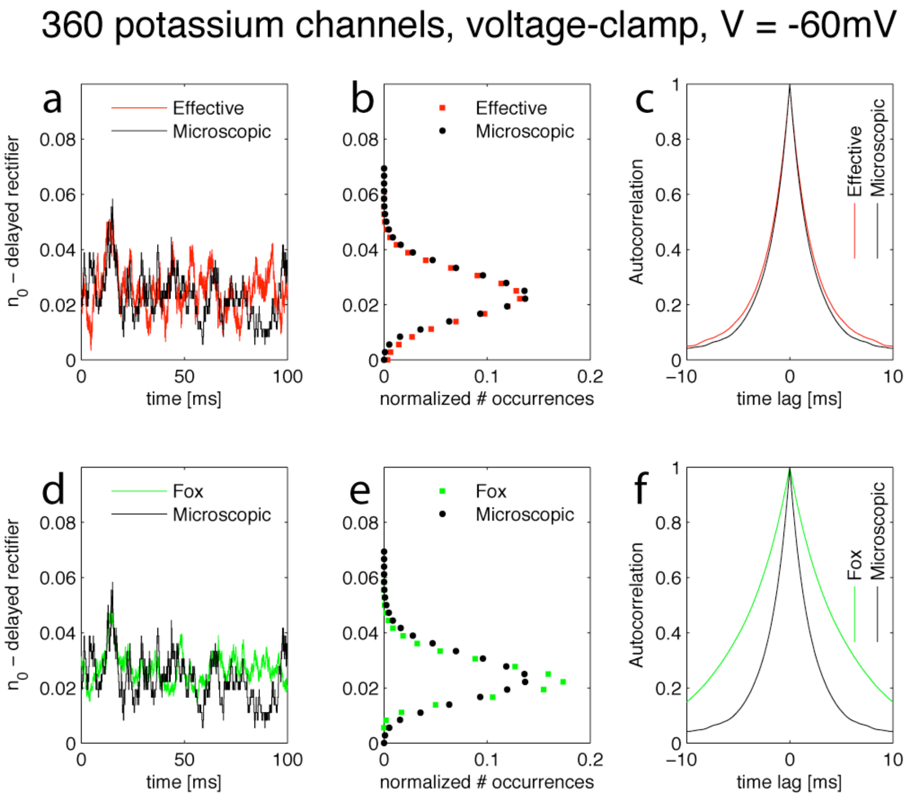

Figure 5: As Figure 3A-C and 3G-I in the Main Text, but for a different value of the holding membrane potential.

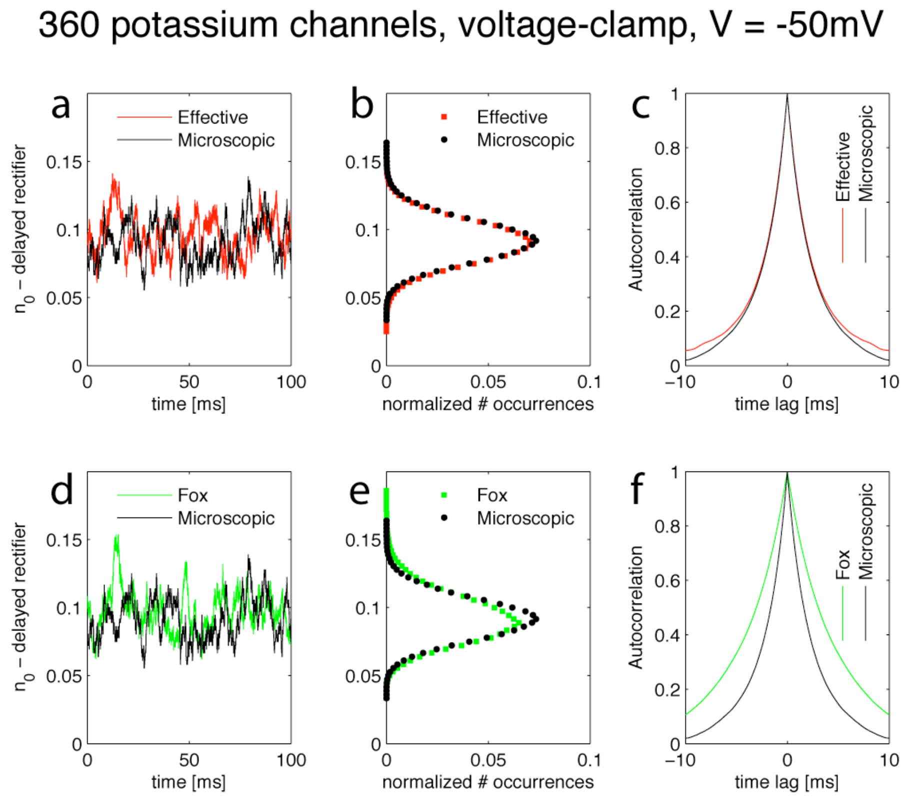

Figure 6: As Figure 3A-C and 3G-I in the Main Text, but for a different value of the holding membrane potential.

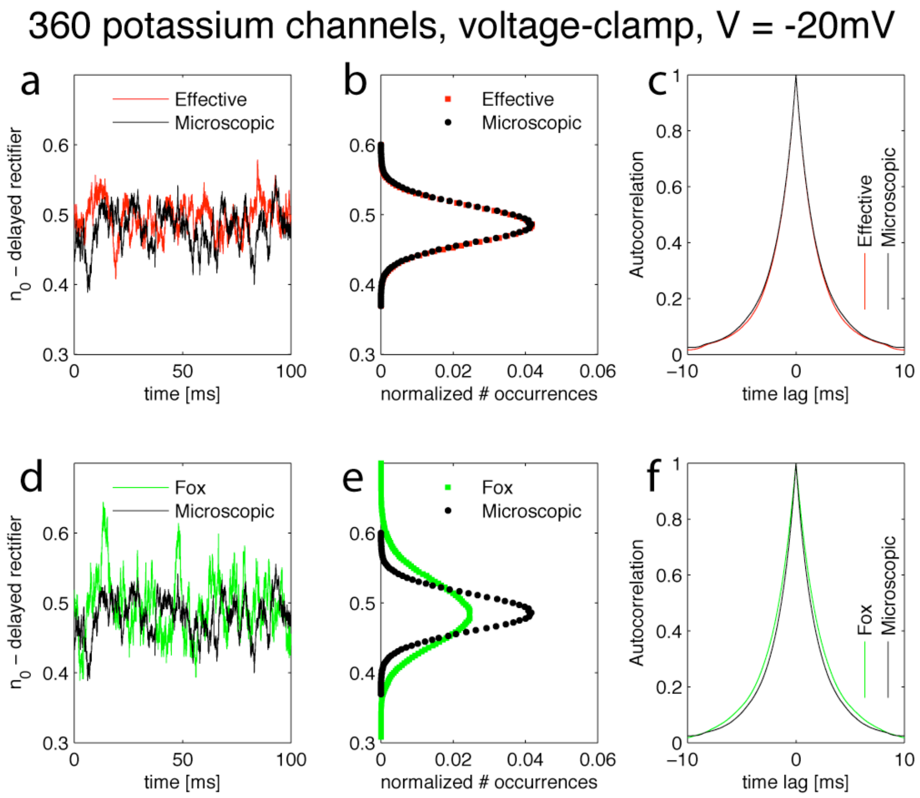

Figure 7: As Figure 3A-C and 3G-I in the Main Text, but for a different value of the holding membrane potential.

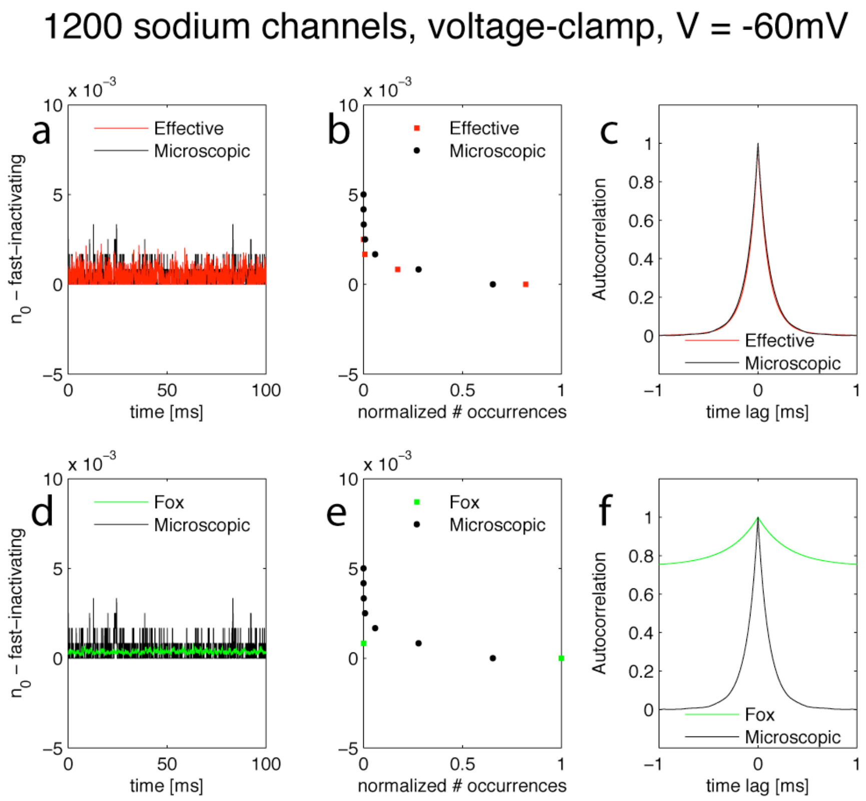

Figure 8: As Figure 3D-F and 3J-L in the Main Text, but for a different value of the holding membrane potential.

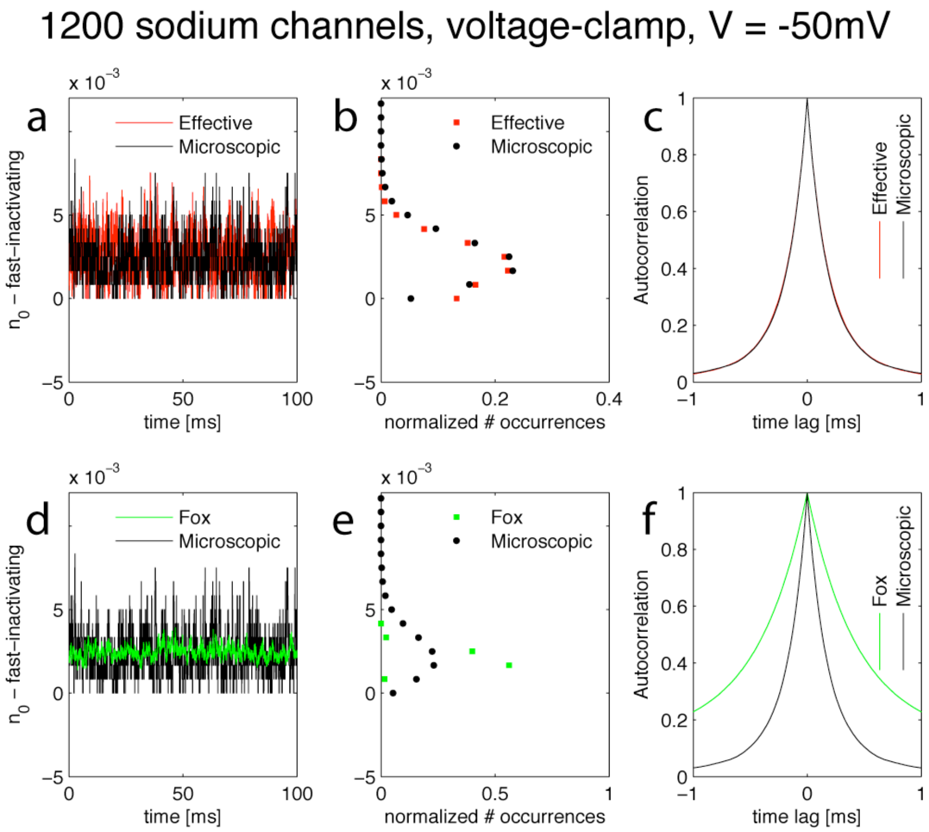

Figure 9: As Figure 3D-F and 3J-L in the Main Text, but for a different value of the holding membrane potential.

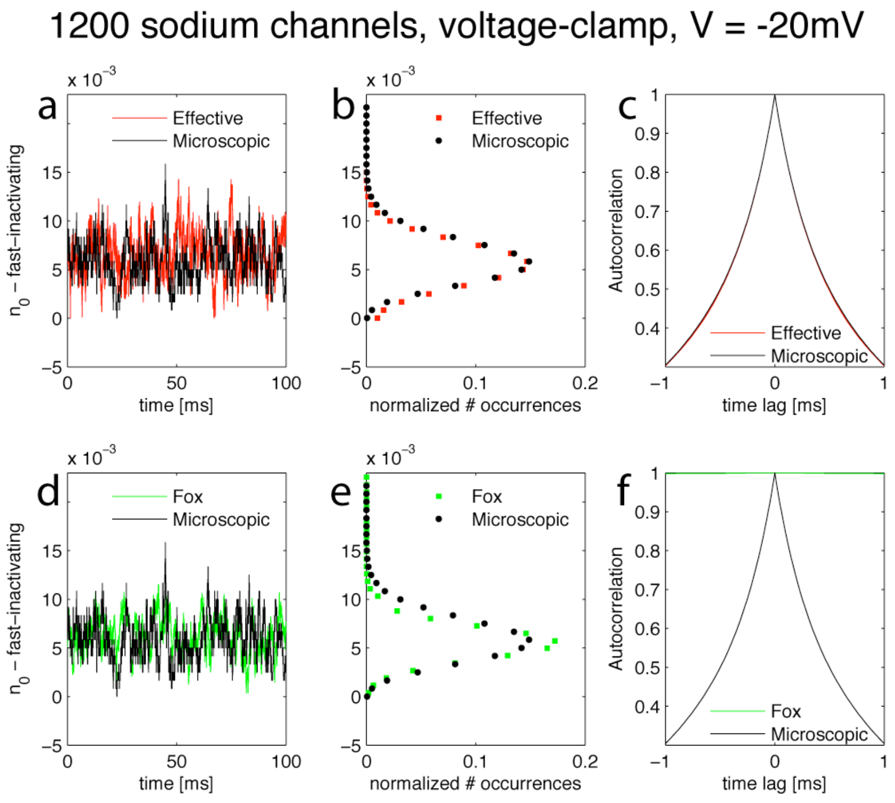

Figure 10: As Figure 3D-F and 3J-L in the Main Text, but for a different value of the holding membrane potential.

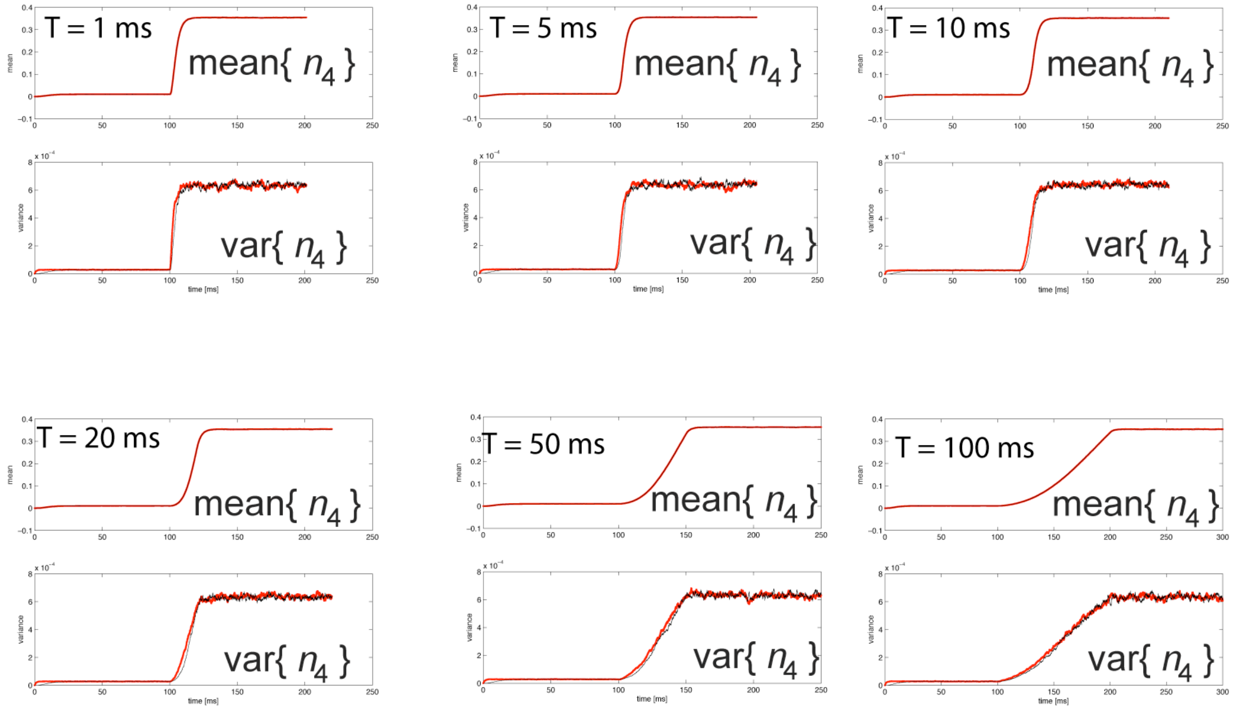

Figure 11: As Figure 3A, under voltage-clamp conditions with the holding membrane potential ramping from *-65mV* to *-30mV* over a time interval T, indicated in each panel. The ramp is started *100ms* after (i.e., approximately at the center of each panel) and its slope varies from *0.35mV/ms* to *35mV/ms*, depending on the value of T (i.e., ranging from *1ms* to *100ms*). The same protocol was repeated for 3000 trials, and both the instantaneous mean and variance of the fraction of open potassium delayed-rectifier channels was computed across time for the microscopic exact Markov model (black traces) and for our effective diffusion approximation (red traces). Both mean and variance are non-stationary under these stimulation protocol conditions, and the time-course of the mean is undistinguishable in the two models (see Eqs. 6, 14). The instantaneous variance resulting from the effective approximation however slightly anticipate the profile obtained from the exact model.

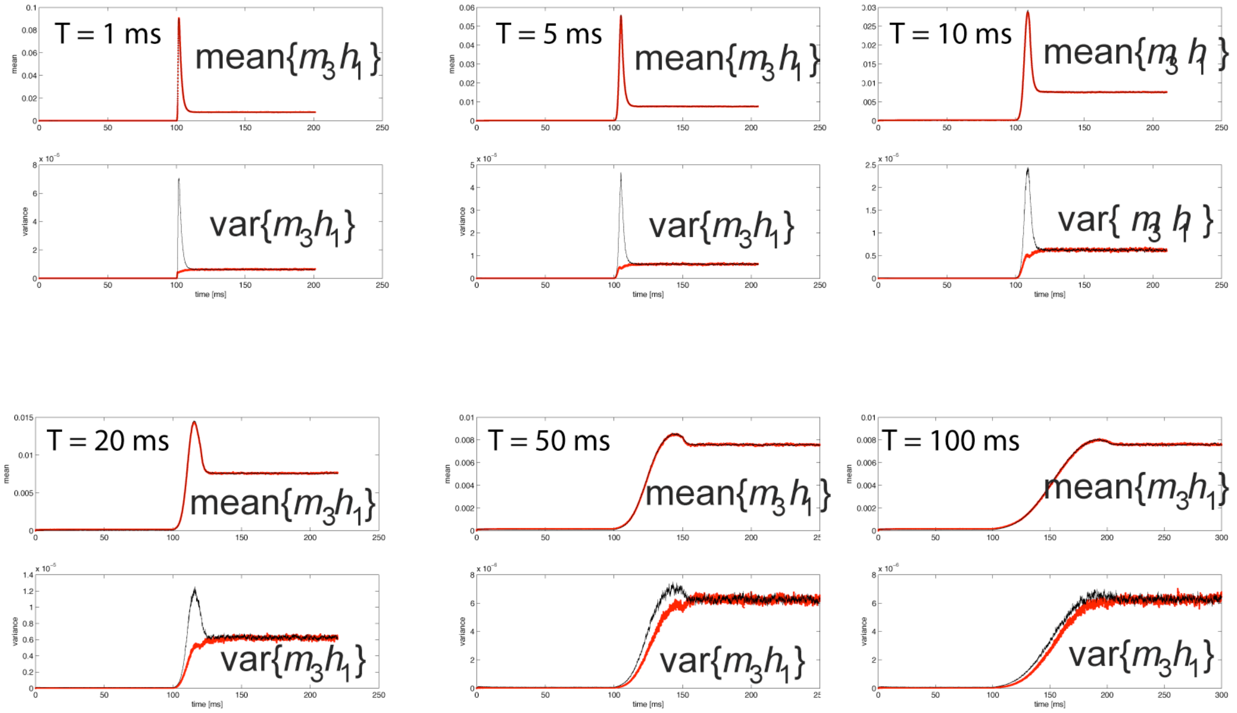

Figure 12: As Figure 3D, under voltage-clamp conditions with the holding membrane potential ramping from *-65mV* to *-30mV* over a time interval T, indicated in each panel. The ramp is started *100ms* after (i.e., approximately at the center of each panel) and its slope varies from *0.35mV/ms* to *35mV/ms*, depending on the value of T (i.e., ranging from *1ms* to *100ms*). The same protocol was repeated for 3000 trials, and both the instantaneous mean and variance of the fraction of open sodium fast-inactivating channels was computed across time for the microscopic exact Markov model (black traces) and for our effective diffusion approximation (red traces). Both mean and variance are non-stationary under these stimulation protocol conditions, and the time-course of the mean is undistinguishable in the two models (see Eqs. 6, 14). The instantaneous variance resulting from the effective approximation however lags behind the very fast changes in the profile obtained from the exact model.

Supplemental References

Klugman SA, Panjer HH, Willmot GE (2008) Loss Models: From Data to Decisions, 3rd edition, Wiley & Sons, Chapter 5, pp. 61-3.
